# Attenuation of lymphocyte immune responses during *Mycobacterium avium* complex-induced lung disease due to increasing expression of programmed death-1 on lymphocytes

**DOI:** 10.1038/srep42004

**Published:** 2017-02-07

**Authors:** Chin-Chung Shu, Jann-Yuan Wang, Ming-Fang Wu, Chen-Tu Wu, Hsin-Chih Lai, Li-Na Lee, Bor-Luen Chiang, Chong-Jen Yu

**Affiliations:** 1 Graduate Institute of Clinical Medicine, College of Medicine, National Taiwan University, Taipei, Taiwan; 2 Department of Traumatology, National Taiwan University Hospital, Taipei, Taiwan; 3 Department of Internal Medicine, National Taiwan University Hospital, Taipei, Taiwan; 4 Genomics Research Center, Academia Sinica, Taipei, Taiwan; 5 Graduate Institute of Toxicology, College of Medicine, National Taiwan University, Taipei, Taiwan; 6 Department of Pathology, National Taiwan University Hospital, Taipei, Taiwan; 7 Department of Medical Biotechnology and Laboratory Science, Chang Gung University, Tao-Yuan, Taiwan; 8 Department of Laboratory Medicine, and National Taiwan University Hospital, Taipei, Taiwan; 9 Department of Pediatrics, National Taiwan University Hospital, Taipei, Taiwan

## Abstract

*Mycobacterium avium* complex-induced lung disease (MAC-LD) becomes important due to its increasing prevalence. Attenuated cellular immunity associated with programmed cell death (PD)–1 may play a pathophysiological role in MAC-LD but lacks of investigation. We enrolled 80 participants in this prospective study, including 50 with MAC-LD and 30 healthy controls. Peripheral blood mononuclear cells (PBMCs), lymphocytes and monocyte-derived macrophages were used for MAC antigen stimulation. Patients with MAC-LD had lower tumor necrosis factor-α and interferon-γ responses compared to the healthy controls in PBMC stimulation assays with MAC bacilli. These responses improved after MAC treatment. The PD-1 and PD ligand expressions and apoptosis were higher in the lymphocytes of the patients with MAC-LD compared to the controls. Both PD-1 and apoptosis on T lymphocytes were significantly increased in the patients with MAC-LD, either by direct MAC stimulation or by MAC-primed macrophage activation. Partially blocking PD-1 and the PD ligand with antagonizing antibodies in the stimulation assay significantly increased the cytokine production of IFN-γ and decreased the apoptosis on T lymphocytes. In conclusion, the patients with MAC-LD have attenuated lymphocyte immunity, which might be associated with increasing activation of PD-1 and PD-1 ligand. Regulating such activation might improve the lymphocytic secretion of IFN-γ and reduce apoptosis.

Nontuberculous mycobacteria-lung disease (NTM-LD) is an important clinical concern^1^,^2^ because the prevalence of NTM infection has increased over the last ten years^3^,^4^,^5^,^6^. Among NTM infections, *Mycobacterium avium* complex (MAC) is one of the most frequently isolated species responsible for lung disease^3^,^7^,^8^. In fact, MAC ubiquitously exists in the environment, and the clinical significance of a positive sputum culture is only around 35–42%^9^,^10^. Thus, its development is indicative of vulnerability^11^,^12^.

Recent studies have reported reduced interferon-gamma (IFN-γ) responses from whole blood co-cultured with MAC antigen^13^, and from peripheral blood mononuclear cells (PBMCs) stimulated by mitogen plus interleukin-12^14^ or by MAC in patients with NTM-LD^15^. The mechanism responsible for the reduced PBMC response to MAC antigen is not fully understood. Programmed cell death-1 (PD-1) is a negative co-receptor for T cell activation, and it sends inhibitory signals to control the inflammation threshold for antigen stimulation^16^. Once PD-1 is over-expressed in patients with chronic mycobacterial lung disease, apoptosis will increase and cellular immunity will be attenuated^17^,^18^,^19^. Under such conditions of suppressed immunity, MAC bacilli may progress to infection as they enter the airway^20^. However, the PD-1 pathway in the pathogenesis of MAC-LD has yet to be investigated. Therefore, the aim of this study was to investigate the pathogenic role of PD-1 in MAC-LD.

## Results

We enrolled 80 participants, including 50 patients with MAC-LD and 30 healthy controls. The mean age and body mass index (BMI) of the patients with MAC-LD were 63.8 years and 20.6, respectively, and 36% were male (Table 1). Three had autoimmune diseases, including two with autoimmune thyroiditis and one with Sicca syndrome. In terms of other clinical characteristics, 26% of the patients with MAC-LD had a prior history of tuberculosis (TB), and cough was the most common presenting symptom. The patients with MAC-LD had an average 3.1 sets of positive sputum cultures for MAC, 1.1 sets of positive acid-fast stains, and 3.3 chest X-ray (CXR) score. With regards to pulmonary function, the patients with MAC-LD had a relatively low forced expiratory volume in one second/forced vital capacity (69%), and a forced expiratory flow between 25% and 75% of vital capacity (57.9%).

### Attenuated cytokine production in MAC-stimulated PBMCs in the patients with MAC-LD

**Figure 1:**
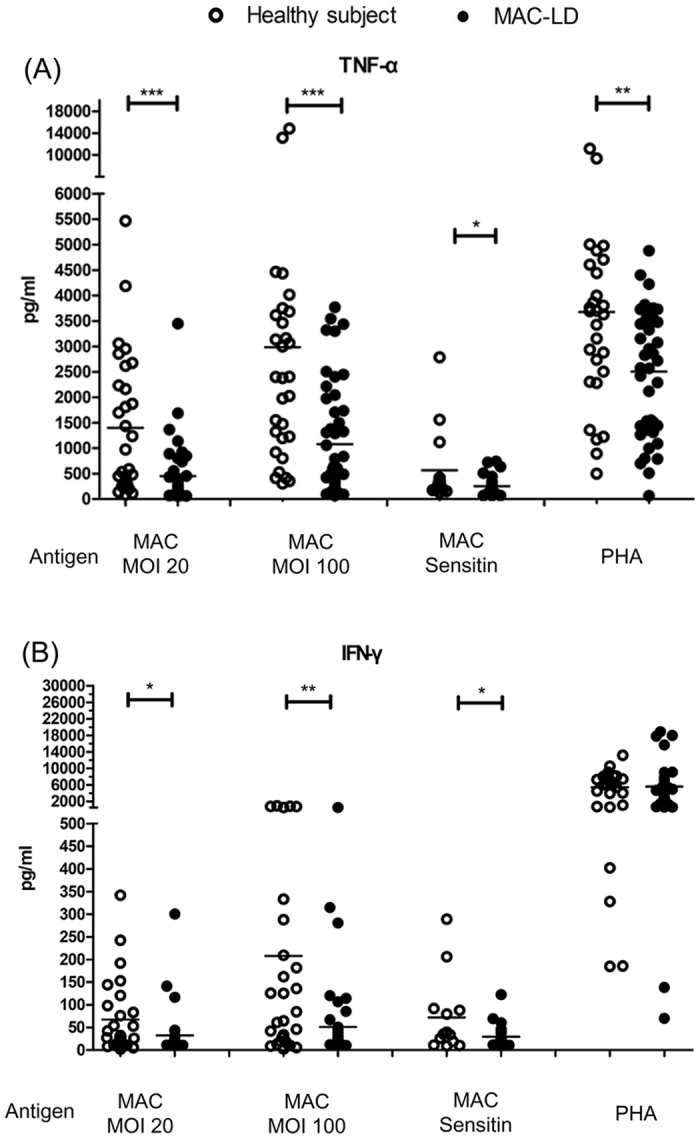
Cytokine responses were measured by assaying peripheral blood mononuclear cells for 48 h with heat-killed *Mycobacterium avium* complex (MAC) bacilli (multiplicity of infection [MOI]: 20 and 100), MAC sensitin (10 μg/ml), and phytohemaglutinin-L (PHA) (2.5 ng/ml) in patients with MAC-lung disease (LD) and controls. The samples from all of the enrolled subjects underwent MAC MOI100 and PHA stimulation, and the number of samples assayed for other antigens are described in the Supplement file. Values for (**A**) tumor necrosis factor-alpha (TNF-α) and (**B**) interferon-gamma (IFN-γ) are presented as dot plots with crossed lines of mean values, and were analyzed using the Student’s *t* test. *0.01 ≤ *p* < 0.05; **0.001 < *p* < 0.01; ***p < 0.001.

We stimulated PBMCs with heat-killed *M. avium* bacilli and MAC sensitin and compared the results to phytohemaglutinin-L (PHA) which was used as the positive control antigen for lymphocyte stimulation (Fig. 1). The PBMC responses of tumor necrosis factor (TNF)-α were higher in the healthy controls than in the patients with MAC-LD after stimulation with MAC bacilli at a multiplicity of infection (MOI) of 20 (mean ± standard deviation [SD]: 1401.8 ± 1370.3 vs. 449.7 ± 603.7 pg/ml, p = 0.001), MOI = 100 (2982.4 ± 3268.2 vs. 1079.0 ± 1095.5 pg/ml, p = 0.004), and sensitin (10 μg/ml) (567.0 ± 189.0 vs. 255.6 ± 192.1 pg/ml, p = 0.042) (Fig. 1A). The IFN-γ responses to MAC bacilli (MOI = 20 and 100) and MAC sensitin were also higher in the controls than in the patients (all p < 0.05) (Fig. 1B). The IFN-γ response to stimulation by MAC bacilli (MOI = 100) was 207.9 ± 281.2 pg/ml in the controls compared to 51.0 ± 88.7 pg/ml in the patients (p = 0.007). However, even though the TNF-α response was different between the healthy controls and patients with MAC-LD with PHA stimulation, there was no difference in IFN-γ response (p = 0.856). This result may suggest that susceptibility to MAC is caused by attenuated IFN-γ production.

There was no significant difference in gender between the control and patient groups (36% vs. 33% male, p = 0.809, chi square test), but the controls were significantly younger than the patients (36.8 vs. 63.8 years, p < 0.001, Student’s *t* test). The cytokine production in the PBMC assay was similar between the participants aged ≥ 65 years and those aged < 65 years (IFN-γ under MAC MOI = 100: 101.7 ± 243.0 vs. 110.6 ± 178.0 pg/ml, p = 0.955 [Table E1, Supplement File]). In addition, we compared 15 age- and sex-matched pairs of patients and controls to validate the IFN-γ responses from the PBMCs stimulated by MAC, and the results still showed a lower response in the MAC-LD group (286.3 ± 304.8 vs. 96.3 ± 138.1 pg/ml, p = 0.020, Mann Whitney *U* test [Fig. E1, Supplement File]).

### Potent PD-1 expression and enhanced apoptosis on T lymphocytes in the patients with MAC-LD

**Figure 2:**
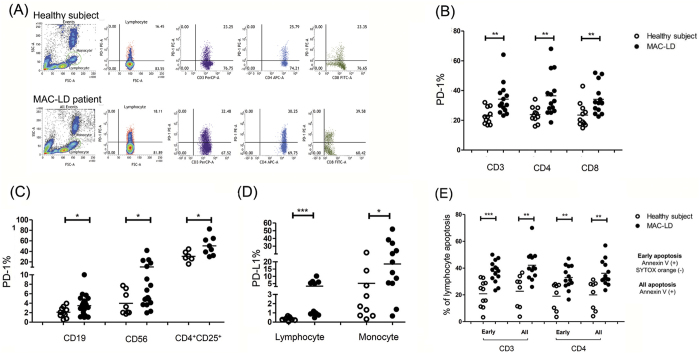
Programmed cell death-1 (PD-1), PD ligand-1 (PD-L1), and apoptosis expressions in peripheral blood lymphocytes and monocytes were measured by flow cytometry. The results shown are (**A**) case demonstration and (**B**,**C**,**D** and **E**) dot plots between the controls and patients with *Mycobacterium avium* complex-lung disease (MAC-LD). We discriminated the lymphocytes (red circles) and monocytes (green circles) by forward scatter (FSC) and side scatter (SSC). We first gated the lymphocyte markers CD3, CD19 and CD56, and CD14 for monocytes. We then gated CD4 and CD8 in CD3-positive lymphocytes and CD25 in CD4-positive lymphocytes. In Figure 2D, we identified monocyte by CD14 positive cells in the monocyte population whereas lymphocyte population was defined by FSC and SSC (Fig. 2D). The crossed lines in the dot plots (B-E) are mean values. The data were compared using the Mann Whitney *U* test. *0.01 ≤ *p* < 0.05. Apoptosis was assessed by Annexin V and SYTOX orange staining. Early apoptosis was defined as Annexin V (+) and SYTOX orange (−), while overall apoptosis was defined as Annexin V (+) regardless of the SYTOX orange results.

To further investigate whether PD-1/PD-L1 was responsible for the low IFN-γ response in the PBMCs of the patients with MAC-LD, we measured the expressions of PD-1 and PD-L1 on PBMCs using flow cytometry. The results showed that the expression of PD-1 was higher in CD3 (mean ± SD: 34.2 ± 10.8% vs. 22.8 ± 5.4%, p = 0.003), CD4 (36.5 ± 14.1% vs. 23.8 ± 5.3%, p = 0.007), CD8 (34.1 ± 9.9% vs. 23.5 ± 8.3%, p = 0.008), CD19 (3.5 ± 2.2% vs. 2.1 ± 1.1%, p = 0.029), CD56 (10.4 ± 10.5% vs. 3.9 ± 2.5%, p = 0.028), and CD4^+^CD25^+^cells (50.4 ± 18.1% vs. 30.5 ± 9.9%, p = 0.023) of the patients with MAC-LD compared to the healthy controls (Fig. 2A–C, Mann Whitney *U* test). In a comparison with six age-matched controls, the patients still had a higher PD-1 expression (Fig. E2, Supplement File). The PD-1 expression in CD14^+^monocytes was similarly low between the controls and patients (1.8 ± 0.9% vs. 1.5 ± 1.0%, p = 0.403). In contrast, the patients had a higher PD-L1 expression on lymphocytes (3.3 ± 3.2% vs. 0.4 ± 0.2%, p < 0.001) and monocytes (18.5 ± 16.1% vs. 5.2 ± 7.5%, p < 0.05) than the controls (Fig. 2D). There were no significant differences in cell sub-populations of the PBMCs between the controls and patients in terms of CD3, CD4, CD8, CD19, or CD14 (Fig. E3, Supplement File).

In addition to PD-1 expression, the apoptosis status of T lymphocytes was measured by Annexin V and SYTOX orange staining. MAC-LD status was associated with higher early (Annexin V [ + ], and SYTOX orange [-]) (32.9 ± 8.7%) and late apoptosis status (all Annexin V [ + ]) (36.1 ± 9.4%) on CD4 lymphocytes compared to the healthy controls (19.0 ± 10.2%, p = 0.003; and 20.0 ± 10.9%, p = 0.001, respectively) (Fig. 2E). The trend of apoptosis on CD3 lymphocytes was also higher in the patient group. The apoptosis status on CD8 was not measured in the present study.

### Changes in cytokine production and PD-1 expression after anti-MAC treatment

**Figure 3:**
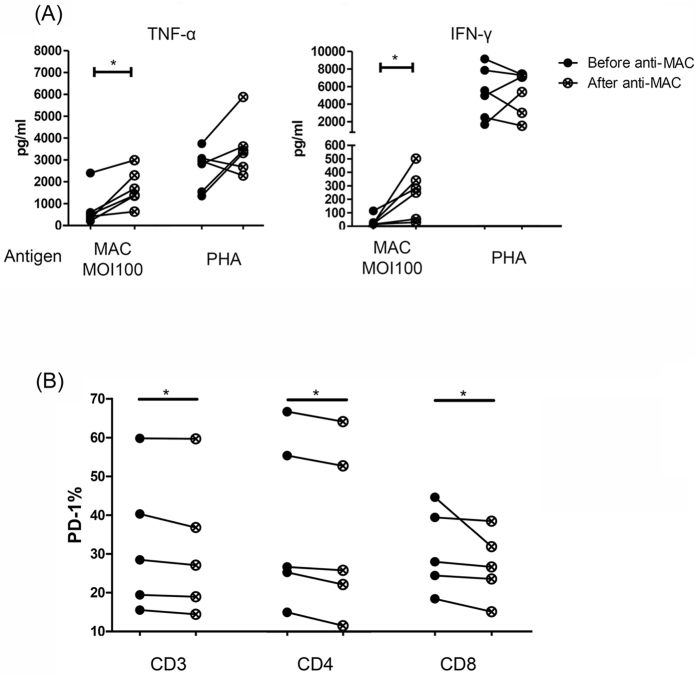
(**A**) Cytokine response (n = 6) and (**B**) programmed cell death-1 (PD-1) expression (n = 5) were measured before and after 2 months of treatment for *Mycobacterium avium* complex lung disease (MAC-LD), and intra-subject changes were compared using the Wilcoxon test. Cytokine responses were measured by assaying peripheral blood mononuclear cells for 48 h with heat-killed MAC bacilli (multiplicity of infection: 100), and phytohemaglutinin-L (PHA) (2.5 ng/ml) in patients with MAC-lung disease. The percentage of PD-1 is shown in the before-and-after graph with intra-subject comparisons. The average PD-1 expression on CD3, CD4, and CD8 decreased from 32.7 ± 17.9% (mean ± standard deviation), 37.9 ± 22.2%, and 31.2 ± 11.2%, to 31.4 ± 18.0%, 35.4 ± 22.3%, and 27.3 ± 8.9%, respectively (all p < 0.05, Wilcoxon test). *0.01 ≤ *p* < 0.05. TNF-α, tumor necrosis factor-alpha; IFN-γ, interferon-gamma.

To investigate the effect of anti-MAC treatment, we measured the cytokine response from the PBMC stimulation assay before and after 2 months of treatment for MAC-LD. The trend of cytokine response was significantly increased after the treatment, including TNF-α (from 741.0 ± 825.4 to 1726.1 ± 820.6 pg/ml, p = 0.031) and IFN-γ (from 35.0 ± 39.1 to 242.5 ± 178.7 pg/ml, p = 0.031, Wilcoxon test), while the differences with PHA stimulation were not significant (Fig. 3A). The cytokine responses improved after MAC treatment, possibly suggesting that the suppressed PBMC function might be due to chronic MAC infection. The PD-1 expression decreased slightly after 2 months of anti-MAC treatment (Fig. 3B and Fig. E8 in the Supplement file). The expression of PD-1 on CD3, CD4, and CD8 decreased from 32.7 ± 17.9%, 37.9 ± 22.2%, and 31.2 ± 11.2%, to 31.4 ± 18.0%, 35.4 ± 22.3%, and 27.3 ± 8.9%, respectively (all p < 0.05, Wilcoxon test).

### MAC stimulation enhanced PD-1 expression and apoptosis on T lymphocytes

**Figure 4:**
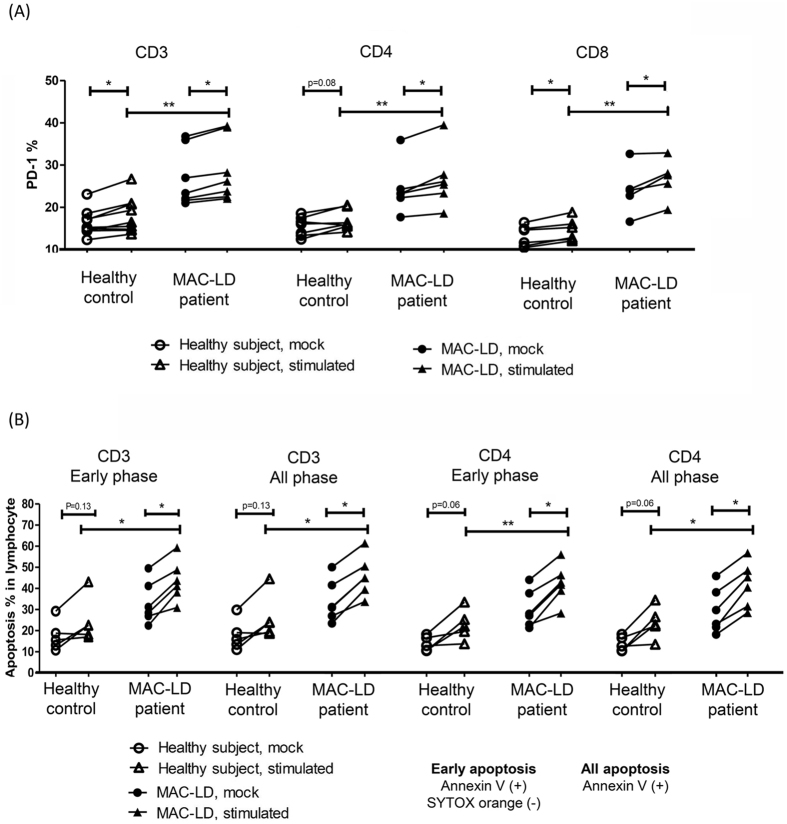
(**A**) Programmed cell death-1 (PD-1) expression and (**B**) apoptosis were assessed by Annexin V and SYTOX orange staining using flow cytometry after 48 h of stimulation of peripheral blood mononuclear cells by multiplicity of infection (MOI) 100 of *Mycobacterium avium* complex (MAC) dead bacilli. Early apoptosis was defined as Annexin V (+) and SYTOX orange (−), whereas overall apoptosis was defined as Annexin V (+) and either SYTOX orange (+) or (−). The Mann-Whitney *U* test was used to compare healthy controls and patients with MAC-lung disease, while the Wilcoxon test was used to compare mock and stimulation in the same subject. *0.01 ≤ *p* < 0.05; **0.001 < *p* < 0.01; ***p < 0.001.

To assay the cell response to MAC, the PBMCs were stimulated *in vitro* with heat-killed MAC bacilli for 48 h. The PD-1 expression and apoptosis status were then assessed on T lymphocytes (Fig. 4). The apoptosis with mock stimulation seemed to be lower than the baseline level, which may have been due to different time points and inter-subject variation. Thus, mock- and MAC-stimulation at the same time point (after 2 days of assay) were compared in the same subjects. With regards to the PBMCs with MAC stimulation, the increases in PD-1 expression and apoptosis were statistically significant in CD3, CD4, CD8 lymphocytes by one-way ANOVA (all p < 0.001). For CD4 lymphocytes, MAC stimulation significantly increased the expression of PD-1 (from 24.4 ± 6.1% to 26.7 ± 7.0%, p = 0.03) (Fig. 4A) and apoptosis (early phase: from 30.0 ± 8.9% to 42.1 ± 9.1%; all phases: from 29.2 ± 10.7% to 41.6 ± 10.6%, both p < 0.05, Wilcoxon test) (Fig. 4B) in the patients with MAC-LD. Compared to the healthy controls under MAC stimulation, the PD-1 expression and apoptosis were higher in lymphocytes from the patients (p < 0.01 for PD-1% and p < 0.05 for apoptosis). A similar trend was also observed in CD3 subsets of T lymphocytes.

### PD-1 and PD-L1 interaction in MAC-LD contributed to cytokine production and apoptosis

**Figure 5:**
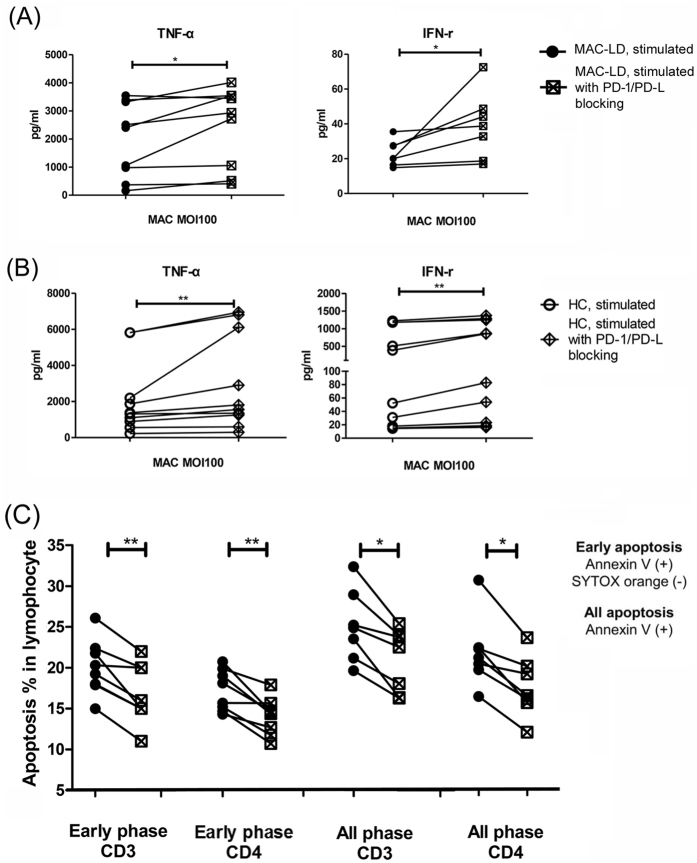
The effect of blocking programmed cell death-1 (PD-1) and programmed death ligand-1&2 (PD-L1&2) was measured by (**A**) cytokine production in the patients with *Mycobacterium avium* complex-lung disease (MAC-LD), and (**B**) that in the healthy subjects, and by (**C**) apoptosis status in PBMCs from the patients with MAC-LD stimulated using MAC (MOI 100). The Wilcoxon test was used to analyze the assays with or without blocking antibodies. TNF-α, tumor necrosis factor-alpha; IFN-γ, interferon-gamma; *0.01 ≤ *p* < 0.05; **p < 0.01.

Given the higher expression of PD-1/PD-L1 on the lymphocytes of the patients with MAC-LD, and that the PD-1 expression and apoptosis were responsive to MAC stimulation, we investigated whether a high expression of the PD-1 pathway on lymphocytes in the patients with MAC-LD was responsible for the decrease in cytokine production and increase in apoptosis. PBMCs were pre-treated with antagonizing PD-1, PD-L1, and PD-L2 antibodies (10 μg/mL each) (eBioscience, USA) for 1 hour. After washing out the antibodies, the PBMC stimulation assay was conducted with MAC (MOI = 100). Cytokine production significantly increased compared to the PBMCs that were not pre-treated with blocking antibodies (Fig. 5A). The expression of TNF-α increased from 1969.0 ± 1342.9 to 2463.2 ± 1412.6 pg/ml (p = 0.020), and IFN-γ increased from 23.1 ± 7.3 to 39.0 ± 19.1 pg/ml (p = 0.016, Wilcoxon test). The blocking effect of MAC-stimulated PBMCs also existed in the control group (Fig. 5B), and increased the responses of TNF-α (from 2118.4 ± 2034.1 to 2964.1 ± 2627.2 pg/ml) and IFN-γ (from 460.6 ± 533.8 pg/ml to 582.1 ± 595.8 pg/ml) in the PBMC assay (both p < 0.01). The post-blocking cytokine levels were not significantly different between the patients and controls (Fig. E9, Supplement file), and the increase in IFN-γ was similar between the controls and patients (1.4 ± 0.4 vs. 1.7 ± 0.9, p = 0.475, Mann Whitney U test). Flow cytometry demonstrated decreased apoptosis on the pre-treated T lymphocytes in the stimulation assay in the patients with MAC-LD (Fig. 5C). The early phase of apoptosis (Annexin V [ + ] but SYTOX orange [−]) on CD4 lymphocytes decreased from 17.2 ± 2.5% to 14.1 ± 2.3% (p = 0.008, Wilcoxon test), and apoptosis overall decreased from 21.5 ± 4.3% to 17.3 ± 3.7% (p = 0.016). A similar pattern was also observed in CD3 + lymphocytes, suggesting that blocking the PD-1 pathway may improve lymphocyte function and reduce apoptosis.

### MAC-primed macrophages induced PD-1/PD-L1 expression on CD4 lymphocytes

**Figure 6:**
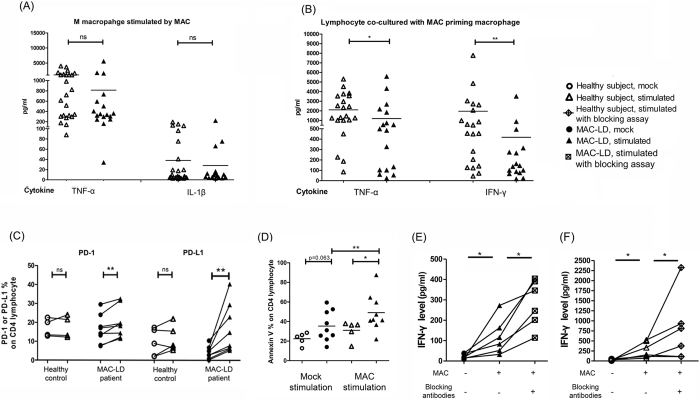
(**A**) Cytokines were measured by assaying human monocyte derived macrophages with heat-killed *Mycobacterium avium* bacilli (MAC [MOI:100]) for 24 h. After co-culturing MAC-primed macrophages with CD14-negative cells from the same subjects for 5 days, we measured (**B**) cytokine responses, (**C**) the expression of programmed cell death-1 (PD-1), PD ligand-1 (PD-L1), and (**D**) Annexin V on CD4 lymphocytes, and changes in (**E**) interferon-gamma (IFN-γ) by blocking PD-1/PD-L1 in the patients with MAC-lung disease (LD) and (**F**) healthy controls. The crossed lines in dot plots (**A**,**B**,**D**) are mean values. The Mann-Whitney *U* test was used to compare controls and patients, while the Wilcoxon test was used to compare the assays with or without blocking antibodies in the same subject. TNF-α, tumor necrosis factor-alpha; IL-1β, interleukin-1 beta; IFN-γ, interferon-gamma; MOI, multiplicity of infection; ns, no statistical significance. *0.01 < *p* < 0.05; **p < 0.01.

To understand the details of the PD-1 pathway in MAC-LD, blood monocyte-derived macrophages were stimulated by MAC bacilli and lipopolysaccharide for 24 h. The cytokine production of TNF-α (1041.2 ± 1044.0 vs. 814.0 ± 1316.0 pg/ml, p = 0.190 Mann Whitney test) and interleukin (IL)−1β (37.9 ± 56.3 vs. 28.0 ± 54.3 pg/ml, p = 0.980) were similar between the healthy subjects and patients with MAC-LD (Fig. 6A). The antigen-primed macrophages were then co-cultured with CD14-negative cells (1:10) from the same patient for 5 days. Cytokine production of TNF-α and IFN-γ from the lymphocytes co-cultured with the MAC-primed macrophages was significantly higher in the healthy subjects than that in patients with MAC-LD (TNF-α: 2101.0 ± 1536.8 vs. 1192.3 ± 1640.1, p = 0.025; and IFN-γ: 1956.0 ± 3130.8 vs. 417.5 ± 856.8 pg/ml, p = 0.0019, Mann Whitney test) (Fig. 6B).

For the co-cultured lymphocytes, we measured the expressions of PD-1 and PD-L1 on CD4 lymphocytes, and found that the expression of PD-1 increased from 16.1 ± 7.1% to 19.3 ± 7.8% (p = 0.009, Wilcoxon test) and PD-L1 increased from 4.2 ± 3.1% to 16.3 ± 12.2% (p = 0.009, Wilcoxon test) in the MAC-LD group (Fig. 6C). The degrees of increase in PD-1 (1.24 ± 0.23 vs. 1.01 ± 0.10 times, p = 0.042, Mann Whitney *U* test) and PD-L1 (8.10 ± 11.9 vs. 1.83 ± 1.22 times, p = 0.011, Mann Whitney *U* test) on the CD4^ + ^lymphocytes were significantly higher in the patients than in the controls. Apoptosis (Annexin V [ + ]) on the CD4 lymphocytes of the patients with MAC-LD became significantly higher than that in the healthy controls after co-cultured with MAC-primed macrophages (49.1 ± 19.0% vs. 31.1 ± 9.7%, p = 0.019, Mann Whitney test), whereas the difference was not significant with mock-primed macrophages (35.4 ± 15.5% vs. 22.5 ± 6.2%, p = 0.063) (Fig. 6D). The expression of Annexin V on the CD4 lymphocytes was significantly increased in the patients with MAC-LD (49.1 ± 19.0% vs. 35.4 ± 15.5%, p = 0.004) but not in the controls (p = 0.134, Wilcoxon test).

We then conducted a blocking assay with antagonizing PD-1 and PD-L1 antibodies to evaluate the role of the pathway. We used PD-L1 antibodies for the MAC-primed macrophages, and treated the CD14-negative cells with PD-1 antibodies for 1 hour before co-culturing the two kinds of cells. The cells were co-cultured for 5 days, after which IFN-γ production was significantly higher in those with PD-1/PD-L1 blocking (284.1 ± 115.5 pg/ml) compared to those without PD-1/PD-L1 blocking (119.9 ± 88.0 pg/ml, p = 0.031, Wilcoxon test) (Fig. 6E). Similarly, the production of IFN-γ increased with PD-1/PD-L1 blocking in the co-culture assay using MAC-primed macrophages from the healthy controls (from 329.4 ± 277.5 to 828.2 ± 758.7 pg/ml, p = 0.039, Wilcoxon test, Fig. 6F). The post-blocking level (Fig. E10, Supplement file) was not significantly different between the controls and patients, and the degree of increase of IFN-γ was similar (2.8 ± 1.9 vs. 3.0 ± 1.2 times, p = 0.852, Mann Whitney U test).

The co-cultured cells were re-stimulated with anti-CD3 (5 μg/ml) and anti-CD28 (1 μg/ml) antibodies for 1 day. We then performed intracellular IFN-γ staining and measured the expression of PD-1, which was higher on the CD4^+^IFN-γ^+^lymphocytes in the patients than in the controls (65.7 ± 17.2% vs. 44.4 ± 19.4%, p = 0.044) (Fig. E11B, Supplement file). After performing the blocking assay, the expressions of PD-1 and PD-L1 on the CD4 lymphocytes decreased from 35.5 ± 24.6% to 33.0 ± 24.9% (p = 0.041, Wilcoxon test) and 22.0 ± 21.9% to 7.8 ± 7.7% (p = 0.021, Wilcoxon test), respectively (Fig. E11C, Supplement file). The percentages of change were 7.04% and 64.54% for PD-1 and PD-L1, respectively.

## Discussion

In the present study, the patients with MAC-LD had a weak *in vitro* PBMC response to *M. avium* bacilli and sensitin, and this response was lower than that of the controls. In addition, the expressions of PD-1 and PD-L1 and apoptosis on the T lymphocytes were higher in the patients, and could be induced directly by MAC bacilli stimulation or indirectly by MAC-primed macrophages. The cytokine production in the PBMC assay and lymphocyte activation assay was improved by blocking PD-1 and PD-1 ligand.

The burden of MAC-LD has increased in the last decade, however the causes remain unclear^3^,^6^,^21^. While the common hypothesis is an increase in the number of immuno-compromised patients, the role of the host and interactions with MAC also need to be clarified^7^,^11^,^22^,^23^. The patients with MAC-LD in the present study had lower PBMC responses of both TNF-α and IFN-γ to *M. avium* bacilli stimulation compared to the healthy controls. However, the IFN-γ response level to PHA stimulation was similar, indicating that chronic MAC infection may induce suppression of cellular immunity. In the macrophage experiment, cytokine production was similar to macrophage stimulation by MAC, but different in lymphocyte activation by MAC-primed macrophages. This suggests that lymphocyte activation may be a key step in attenuating MAC-associated immunity.

The exact mechanism of action of *M. avium* bacilli is unclear. Our results suggest the enhanced exhaustion of PD-1 and PD-L1 on lymphocytes and PD-L1 on monocytes in MAC-LD, which then attenuates the activation of immunity. In a previous study, the PD-1 pathway was shown to suppress T cell function and increase apoptosis in a model of *Mycobacterium tuberculosis* infection^24^,^25^. In addition, the expression of PD-1 has been reported to be higher in patients with TB on CD3, CD4, CD8, and CD19-positive lymphocytes^25^, similar to the results of our patients with MAC-LD. In contrast, the expression of PD-1 was enhanced on CD14^+^monocytes in the patients with TB^25^, whereas there was no corresponding increase in our patients with MAC-LD.

The expression of natural killer (NK) cells has been reported to be impaired with decreased IFN-γ production and lytic degranulation by the PD-1 pathway in *Mycobacterium tuberculosis* infection^26^,^27^. The PD-1 expression on NK cells was higher in the patients with MAC-LD than in the controls. On the other hand, CD4^+^CD25^+^lymphocytes in patients with MAC-LD may be considered as regulatory T cells and have an over-expression of PD-1 and population expansion, which is similar to patients with TB^28^. These observations could also be responsible for the inhibition of immunity by lymphocytes in MAC-LD^19^,^20^,^28^,^29^. As we did not perform intracellular staining for cytokines in the present study, we could not ascertain the lymphocyte cell types contributing to the increase in cytokines after stimulation or blockade.

In the present study, blocking the PD-1 and PD-1 ligands under MAC stimulation augmented IFN-γ production and reduced lymphocyte apoptosis, consistent with previous reports on TB^25^,^26^,^30^. An increase in IFN-γ with the blocking assay was noted in both the patients with MAC-LD and controls in this study. However, several studies on sarcoidosis and pulmonary TB showed no effect of blocking PD-1 in healthy controls^25^,^31^. There are several possible explanations for this finding. First, responses to blocking the PD-1 pathway might differ between different diseases and different pathogens. For example, Kartalija *et al* reported that IFN-γ production from PBMCs in response to MAC bacilli was weaker in patients with MAC-LD than in healthy controls^13^, which is consistent with the findings of the present study but in contrast to patients with TB who have been reported to have higher IFN-γ production than controls during stimulation with *Mycobacterium tuberculosis* antigens^32^. Second, the preparation of MAC bacilli in the present study may not have been sufficiently pure, which may have resulted in nonspecific responses. Third, there were differences in measurements among the studies. For example, Braun *et al*^31^ measured lymphocyte proliferation after blocking PD-1/PD-1 ligands on PBMCs under T cell receptor stimulation (anti-CD3 and anti-CD28 antibodies), whereas in the present study we measured secreted cytokines under MAC stimulation. On the other hand, Singh *et al*^25^ reported no obvious increase in IFN-γ^+^CD3^+^T cells in PPD-positive healthy controls after the blocking assay, whereas we measured total soluble cytokine production in culture supernatant but not in specific T cell subsets. Other immune cells may produce IFN-γ against infection^33^,^34^. Therefore, further studies are needed to elucidate the reasons for the differences in results in healthy subjects between the present study and other studies.

In this study, we observed that an increase in PD-1 was induced by MAC stimulation in both the patients with MAC-LD and healthy controls (Fig. 4), and the effect of the blocking assay might have been through suppressing the pathway activated by MAC. We hypothesize that the increase in the PD-1 pathway under MAC stimulation may not be the cause of MAC-LD but rather a presentation of immune exhaustion during chronic MAC-LD. In addition, the effect of the PD-1 blocking assay does not necessarily mean that healthy subjects will be more prone to MAC-LD, because the blocking effect may only be due to manipulation of the immunomodulation induced by MAC, and other host susceptibilities exist for MAC-LD which were not investigated in the present study^35^.

In patients with *M. tuberculosis* infection, the expressions of PD-1 and PD-ligands have been reported to be lower after anti-TB treatment, suggesting that the PD-1 pathway is induced by mycobacterial infection^25^,^36^. We also found a decreasing trend of PD-1 expression on lymphocytes after 2 months of treatment for MAC-LD. However, the degree of the decrease in PD-1 expression after anti-MAC treatment was not as pronounced as in previous reports in patients with TB^25^. The differences between TB and NTM are unclear, but might involve difficulty in treating MAC-LD or a more chronic course of NTM-LD^37^,^38^. Therefore, as MAC-LD is a chronic and relapsing disease^39^,^40^, targeted immune therapy of the PD-1 pathway may be an option worth investigating.

Despite these findings, totally blocking the function of the PD-1 pathway is not beneficial according to previous reports, in which a knockout mice model showed excessive lung inflammation and poor T cell function as well as poor survival in *M. tuberculosis* infection^41^,^42^. Proposed mechanisms include increased PD-L1 and B7–1 interactions, acting T cell inhibition^43^, and recruitment of regulatory T cells^41^. Similarly, patients receiving anti-PD-1 treatment for cancer have been reported to develop TB, probably echoing the decrease in immunity associated with over-suppressing PD-1^44^. Therefore, adequate regulation of the PD-1 pathway may be more beneficial than totally blocking the function. A recent study also emphasized the physiological function of PD-1 in reducing IFN-γ production from CD4 T cells to prevent lethal disease in patients with TB^45^. In the present study, antagonizing PD-1/PD-ligand antibodies partially blocked the PD-1 pathway and improved cytokine production from lymphocytes. The concept of anti-PD-1 therapy has also been suggested in patients with sepsis, which leads to immune exhaustion by PD-1^46^. Further studies are needed to investigate the optimal regulation of the PD-1 pathway, which may be helpful in the control of mycobacterial lung disease.

It is well known that MAC infects macrophages and activates toll-like receptors^47^,^48^. We also found that the expression of PD-1 ligands was increased in macrophages after MAC bacilli stimulation. Previous investigations have demonstrated that the expression of PD-L1 on antigen presenting cells is controlled by signal transducer and activator of transcription (STAT)1 and STAT3^49^,^50^. PD-1 is induced and increasingly expressed on lymphocytes with the upregulation of PD-L1, thereby suppressing T cell function and inducing apoptosis. The c-Jun N-terminal kinase and extracellular signal-regulated kinase pathways have also been reported to be responsible for PD-1 upregulation^51^. Further studies of the intra-cellular pathway in MAC-LD are important to develop new treatment strategies for patients with new macrolide resistance or in refractory cases^18^,^52^.

There are several limitations to the present study. First, the patients and controls were not matched for age. The overall age of the patients with MAC-LD was older than that of the controls which may have led to bias, although we found similar results in age-matched subgroups. Second, an MOI of 100 is high and should be applied cautiously; further validation studies using viable bacilli are needed. Third, we could not identify the intracellular mechanism by which MAC induces the expression of PD-1. Fourth, we used thawed cells which may have resulted in an over-estimation of apoptosis. Fifth, we did not titrate the concentrations of antagonizing antibodies for the PD-1 pathway. In addition, the isotype of the blocking antibody was not controlled, and the blocking effect may have been overestimated. Sixth, we did not perform lymphocyte proliferation assays or intracellular cytokine staining to evaluate lymphocyte function. Finally, we did not investigate *M. avium*-specific CD4 cells in this study, and future studies are warranted.

In conclusion, the lymphocytes of patients with MAC-LD had attenuated function with regards to IFN-γ production and increased apoptosis status, and this may be associated with an increase in the expression of the PD-1 pathway. By partially blocking PD-1 and its ligands, secretion of IFN-γ increased from lymphocytes and apoptosis status improved. Targeted regulation of the PD-1 pathway may have therapeutic potential for MAC-LD in the future, especially for patients who fail current medical treatment.

## Materials and Methods

### Patient enrollment

This prospective study was conducted at National Taiwan University Hospital from April 2012 to July 2016. Patients aged ≥20 years and with respiratory sample(s) that were culture-positive for mycobacteria were screened. Patients with MAC-LD according to the American Thoracic Society (ATS) diagnostic guidelines were enrolled^1^. Healthy subjects without respiratory symptoms and normal chest radiographs were enrolled as the controls. The controls did not receive skin tests using sensitin or purified protein derivatives. Patients with human immunodeficiency virus (HIV) infection, active cancer, concurrent TB and extra-pulmonary NTM infection were excluded.

The Research Ethics Committee of National Taiwan University Hospital approved this study (IRB No: 201012082RC and 201407079RIND). All of the participants provided written informed consent. The methods were carried out in accordance with the approved guidelines.

### Isolation of PBMCs

Ten ml of peripheral blood from the enrolled subjects was sampled in heparin-containing tubes. Mononuclear cells were immediately isolated using Ficoll-Paque PLUS (GE Healthcare Life Sciences, Sweden) and suspended in medium containing RPMI-1640 (Life Technologies; USA), 10% fetal bovine serum (FBS), and 1% penicillin-streptomycin (Life Technologies, USA). Viable cells were counted using a Scepter™ 2.0 Handheld Automated Cell Counter (Millipore Corporation, Billerica, MA, USA). All cells were immediately frozen using CELL-BANKER (ZENOAQ, Japan) following the manufacturer’s instructions. The cells were then stored at −80 °C and defrosted within days for the scheduled experiments.

### Preparation of heat-killed dead *M. avium* bacilli

*Mycobacterium avium* subspecies (American type of culture collection 25291) were cultured in 7H11 solid medium. The bacilli were retrieved after 5 days of culture, and the colonies were counted using the dilution method. A dry bath incubator was used to heat-kill the bacilli at 80 °C for 30 minutes before retrieval of the dead bacilli. The non-viability of the heat-killed bacilli was proven by re-culture for 4 weeks.

### PBMC stimulation assay and PD-1/PD ligand blocking assay

The PBMCs were cultivated in 96-well plates in 300 μl of RPMI 1640 medium with 10% FBS and 1% penicillin-streptomycin (2 × 10^5^ cells per well). Heat-killed *M. avium* bacilli were added with MOI of 0, 20, and 100 for the 2-day co-culture (Fig. E12, Supplement file). MAC sensitin (Statens Serum Institut, Denmark) (10 μg/ml) and lipopolysaccharide (LPS, from *Escherichia coli* 0111:B4) (Sigma-Aldrich, USA) (5 μg/ml) were used as other antigens, as well as PHA (Calbiochem, USA) (2. ng/ml) as the positive control. The reaction supernatant was retrieved for further cytokine assay.

In subsequent studies, antagonistic PD-1 (10 μg/mL), PD-L1 (10 μg/mL), and PD-L2 (10 μg/mL) antibodies (eBiosciences, USA) were reacted with the PBMCs from the patients for 1 hour^25^,^53^. After the antibodies had been washed out, the PBMCs were stimulated using MAC bacilli for 48 h. Cytokine responses and apoptosis were then examined.

### Surface markers and apoptosis expression in different PBMC groups

The components of the PBMCs (1 × 10^6^ cells), including T cells, B cells, NK cells, and monocytes, were measured using flow cytometry (FACSVerse, BD Biosciences, USA) using anti-CD3-PerCP, anti-CD4-APC, anti-CD25-FITC, anti-CD8-FITC, anti-CD14-PerCP, anti-CD19-APC, and anti-CD56-FITC antibodies (BD Biosciences, CA, USA). The PD-1, PD-L1, PD-L2, and apoptosis markers were also stained with anti-PD-1-PE, anti-PD-L1-FITC, anti-PD-L2-APC antibodies (eBiosciences, USA), Annexin V, and SYTOX orange (eBiosciences, USA). Data were analyzed using BD FACSuite V software (BD, Biosciences, USA). We discriminated lymphocyte and monocyte populations using forward scatter (FSC) and side scatter (SSC). We gated the lymphocyte markers CD3, CD19 and CD56 to identify lymphocytes, and CD14 for monocytes. We then gated CD4 and CD8 in CD3-positive lymphocytes and further gated CD25 in CD4-positive lymphocytes. Early apoptosis was defined as Annexin V (+) but SYTOX orange (−), and overall apoptosis was defined as Annexin V (+) regardless of the SYTOX orange results. The PBMCs were stimulated by mock or MAC (MOI 100) for 48 h and stained with PD-1 and T cell and apoptosis markers.

### Macrophage stimulation with subsequent lymphocyte co-culture assay

The CD14^+^monocytes were isolated from PBMCs using a CD14-positive selection system (MACS System, Miltenyi Biotec Inc.) and cultivated in RPMI 1640 medium supplemented with 10% FBS, 50 mM 2-mercaptoethanol (Sigma-Aldrich, USA), and 10 ng/ml recombinant human macrophage colony-stimulating factor (R&D Systems, USA) for 5 days to allow for differentiation into macrophages.

The macrophages were stimulated with medium or MAC (MOI 100) for 48 h, and the expressions of PD-L1 and PD-L2 were then measured (Supplement file). The macrophages were also stimulated with medium, MAC (MOI 20 or 100), and LPS (5 μg/ml) for 24 h. The selected MAC (MOI 100) and mock-stimulated macrophages were then co-cultured with CD14-negative cells from the same subject at a ratio of 1:10 for 5 days. The reaction supernatant was then collected and the co-cultured cells were stained with anti-CD4, anti-PD-1, anti-PD-L1, Annexin V, and SYTOX orange. We performed blocking in the lymphocyte activation assay using antagonistic PD-1 (10 μg/mL) antibodies for CD14-negative cells for 1 hour and PD-L1 (10 μg/mL) antibodies for macrophages before the co-culture in six patients with MAC-LD and eight controls^25^,^53^. We then collected the supernatants for further cytokine assays, and added CD3 (5 μg/ml) and CD28 (1 μg/ml) antibodies (eBioscience, San Diego, CA) to re-stimulate the cells for 24 hours^54^. We added protein transport inhibitor (BD Bioscience, USA) in the second half of the co-culture. We identified CD4^+^IFN-γ^+^ lymphocytes and measured the expression of PD-1 using anti-CD4-APC, anti-IFN-γ-PerCPcy5.5 and anti-PD-1-PE/Cy7.0 (BD Pharmingen, San Diego, CA) in flow cytometry.

### Cytokine examination

All experimental supernatants were stored at −20 °C and examined within 1 month in a random order by a technician blinded to the patients’ clinical diagnosis. The reaction supernatants were tested for IFN-γ for lymphocyte function and TNF-α and IL-1β for pro-inflammatory responses using the a DuoSet ELISA Development System (R&D Systems, USA).

### Data collection and statistical analysis

Clinical data including age, sex, co-morbidities, history of pulmonary TB, and laboratory data at enrollment were recorded. Chest imaging was interpreted as noted in a previous study^7^. Inter-group differences were analyzed using the Student’s *t* test or Mann-Whitney *U* test for numerical variables, where appropriate. The Wilcoxon test was used for paired numerical data in the same subject, especially before and after comparisons or mock vs. antigen comparisons. The chi-square test was used for categorical variables. Statistical significance was set at *p* < 0.05. All analyses were performed using SPSS version 19.0 (SPSS Inc., Chicago, IL).

## Additional Information

**How to cite this article**: Shu, C.-C. *et al*. Attenuation of lymphocyte immune responses during *Mycobacterium avium* complex-induced lung disease due to increasing expression of programmed death-1 on lymphocytes. *Sci. Rep.* **7**, 42004; doi: 10.1038/srep42004 (2017).

**Publisher's note:** Springer Nature remains neutral with regard to jurisdictional claims in published maps and institutional affiliations.

## Supplementary Material

Supplementary Information

## Figures and Tables

**Table 1:** The clinical characteristics of the participants with *Mycobacterium avium* complex-lung disease (MAC-LD).

	MAC-LD n = 50
Age (years)	63.8 [14.0]
Male sex	18 (36%)
Current smoker	4 (8%)
Body mass index, kg/m^2^	20.6 [2.8]
Diabetes mellitus	5 (10%)
Autoimmune diseases^†^	3 (6%)
Prior TB history	13 (26%)
Symptoms	
Cough	44 (88%)
Dyspnea	17 (34%)
Hemoptysis	10 (20%)
Duration, days	763 [1124]
Sputum study within 1 year	
No. of positive AFS	1.1 [1.1]
No. of positive culture	3.1 [1.9]
Radiological finding	
CXR score‡	3.3 [2.4]
Cavitation	4 (8%)
Pulmonary function§	
FVC, % of predicted	93.1 [18.1]
FEV1, % of predicted	79.7 [26.3]
FEV1/FVC, %	69.0 [17.9]
FEF 25–75%, % of predicted	57.9 [36.0]

Abbreviations: AFS, acid-fast smear; CXR, chest radiography; FEF 25–75%, Forced expiratory flow between 25% and 75% of vital capacity; FEV1, Forced expiratory volume in 1^st^ second; FVC, Forced vital capacity; MAC, *Mycobacterium avium* complex; LD, lung disease.

Data are no. (%) or mean [standard deviation]

^†^Autoimmune diseases included two with thyroiditis and one with Sicca syndrome.

^‡^CXR score was interpreted by a total score from six lung zones that contained three respective scores^55^.

^§^Pulmonary function tests were reviewed in 27 patients.

## References

[b1] Griffith D. E. . An official ATS/IDSA statement: diagnosis, treatment, and prevention of nontuberculous mycobacterial diseases. Am J Respir Crit Care Med 175, 367–416 (2007). 17277290 10.1164/rccm.200604-571ST

[b2] Menzies D. & Nahid P. Update in tuberculosis and nontuberculous mycobacterial disease 2012. Am J Respir Crit Care Med 188, 923–927 (2013). 24127799 10.1164/rccm.201304-0687UP

[b3] Lai C. C. . Increasing incidence of nontuberculous mycobacteria, Taiwan, 2000-2008. Emerg Infect Dis 16, 294–296 (2010). 20113563 10.3201/eid1602.090675 PMC2958002

[b4] Field S. K. & Cowie R. L. Lung disease due to the more common nontuberculous mycobacteria. Chest 129, 1653–1672 (2006). 16778288 10.1378/chest.129.6.1653

[b5] Adjemian J., Olivier K. N., Seitz A. E., Holland S. M. & Prevots D. R. Prevalence of nontuberculous mycobacterial lung disease in U.S. Medicare beneficiaries. Am J Respir Crit Care Med 185, 881–886 (2012). 22312016 10.1164/rccm.201111-2016OC PMC3360574

[b6] Marras T. K., Mendelson D., Marchand-Austin A., May K. & Jamieson F. B. Pulmonary nontuberculous mycobacterial disease, Ontario, Canada, 1998-2010. Emerg Infect Dis 19, 1889–1891 (2013). 24210012 10.3201/eid1911.130737 PMC3837646

[b7] Shu C. C. . Clinical characteristics and prognosis of nontuberculous mycobacterial lung disease with different radiographic patterns. Lung 189, 467–474 (2011). 21956280 10.1007/s00408-011-9321-4

[b8] Hoefsloot W. . The geographic diversity of nontuberculous mycobacteria isolated from pulmonary samples: an NTM-NET collaborative study. Eur Respir J 42, 1604–1613 (2013). 23598956 10.1183/09031936.00149212

[b9] Koh W. J. . Clinical significance of nontuberculous mycobacteria isolated from respiratory specimens in Korea. Chest 129, 341–348 (2006). 16478850 10.1378/chest.129.2.341

[b10] van Ingen J. . Clinical relevance of non-tuberculous mycobacteria isolated in the Nijmegen-Arnhem region, The Netherlands. Thorax 64, 502–506 (2009). 19213773 10.1136/thx.2008.110957

[b11] Honda J. R., Knight V. & Chan E. D. Pathogenesis and risk factors for nontuberculous mycobacterial lung disease. Clin Chest Med 36, 1–11 (2015). 25676515 10.1016/j.ccm.2014.10.001

[b12] Chan E. D. & Iseman M. D. Underlying host risk factors for nontuberculous mycobacterial lung disease. Seminars in respiratory and critical care medicine 34, 110–123 (2013). 23460011 10.1055/s-0033-1333573

[b13] Kartalija M. . Patients with nontuberculous mycobacterial lung disease exhibit unique body and immune phenotypes. Am J Respir Crit Care Med 187, 197–205 (2013). 23144328 10.1164/rccm.201206-1035OC PMC5446199

[b14] Kim R. D. . Pulmonary nontuberculous mycobacterial disease: prospective study of a distinct preexisting syndrome. Am J Respir Crit Care Med 178, 1066–1074 (2008). 18703788 10.1164/rccm.200805-686OC PMC2720143

[b15] Vankayalapati R. . Cytokine profiles in immunocompetent persons infected with Mycobacterium avium complex. The Journal of infectious diseases 183, 478–484 (2001). 11133380 10.1086/318087

[b16] Okazaki T., Chikuma S., Iwai Y., Fagarasan S. & Honjo T. A rheostat for immune responses: the unique properties of PD-1 and their advantages for clinical application. Nature immunology 14, 1212–1218 (2013). 24240160 10.1038/ni.2762

[b17] Bhatnagar S. & Schorey J. S. Elevated mitogen-activated protein kinase signalling and increased macrophage activation in cells infected with a glycopeptidolipid-deficient Mycobacterium avium. Cell Microbiol 8, 85–96 (2006). 16367868 10.1111/j.1462-5822.2005.00602.x

[b18] Kitada S. . Long-term radiographic outcome of nodular bronchiectatic Mycobacterium avium complex pulmonary disease. Int J Tuberc Lung Dis 16, 660–664 (2012). 22410245 10.5588/ijtld.11.0534

[b19] Chen L. & Flies D. B. Molecular mechanisms of T cell co-stimulation and co-inhibition. Nat Rev Immunol 13, 227–242 (2013). 23470321 10.1038/nri3405 PMC3786574

[b20] Patsoukis N. . Selective effects of PD-1 on Akt and Ras pathways regulate molecular components of the cell cycle and inhibit T cell proliferation. Science signaling 5, ra46 (2012). 22740686 10.1126/scisignal.2002796 PMC5498435

[b21] Chien J. Y., Lai C. C., Sheng W. H., Yu C. J. & Hsueh P. R. Pulmonary infection and colonization with nontuberculous mycobacteria, Taiwan, 2000-2012. Emerg Infect Dis 20, 1382–1385 (2014). 25062534 10.3201/eid2008.131673 PMC4111185

[b22] Shu C. C. . Use of soluble triggering receptor expressed on myeloid cells-1 in non-tuberculous mycobacterial lung disease. Int J Tuberc Lung Dis 15, 1415–1420 (2011). 22283904 10.5588/ijtld.10.0786

[b23] Dirac M. A. . Environment or host?: A case-control study of risk factors for Mycobacterium avium complex lung disease. Am J Respir Crit Care Med 186, 684–691 (2012). 22859521 10.1164/rccm.201205-0825OC PMC5450977

[b24] Deng R. . B7H1/CD80 interaction augments PD-1-dependent T cell apoptosis and ameliorates graft-versus-host disease. J Immunol 194, 560–574 (2015). 25488990 10.4049/jimmunol.1402157 PMC4282988

[b25] Singh A., Mohan A., Dey A. B. & Mitra D. K. Inhibiting the programmed death 1 pathway rescues Mycobacterium tuberculosis-specific interferon gamma-producing T cells from apoptosis in patients with pulmonary tuberculosis. The Journal of infectious diseases 208, 603–615 (2013). 23661793 10.1093/infdis/jit206

[b26] Alvarez I. B. . Role played by the programmed death-1-programmed death ligand pathway during innate immunity against Mycobacterium tuberculosis. The Journal of infectious diseases 202, 524–532 (2010). 20617899 10.1086/654932

[b27] Kee S. J. . Dysfunction of natural killer T cells in patients with active Mycobacterium tuberculosis infection. Infection and immunity 80, 2100–2108 (2012). 22409933 10.1128/IAI.06018-11 PMC3370582

[b28] Periasamy S. . Programmed death 1 and cytokine inducible SH2-containing protein dependent expansion of regulatory T cells upon stimulation With Mycobacterium tuberculosis. The Journal of infectious diseases 203, 1256–1263 (2011). 21383382 10.1093/infdis/jir011 PMC3069733

[b29] Keir M. E., Freeman G. J. & Sharpe A. H. PD-1 regulates self-reactive CD8 + T cell responses to antigen in lymph nodes and tissues. J Immunol 179, 5064–5070 (2007). 17911591 10.4049/jimmunol.179.8.5064

[b30] Stephen-Victor E. . Inhibition of programmed death 1 ligand 1 on dendritic cells enhances Mycobacterium-mediated interferon gamma (IFN-gamma) production without modulating the frequencies of IFN-gamma-producing CD4 + T cells. The Journal of infectious diseases 211, 1027–1029 (2015). 25258379 10.1093/infdis/jiu532

[b31] Braun N. A. . Blockade of the programmed death-1 pathway restores sarcoidosis CD4( + ) T-cell proliferative capacity. Am J Respir Crit Care Med 190, 560–571 (2014). 25073001 10.1164/rccm.201401-0188OC PMC4214083

[b32] Pai M., Zwerling A. & Menzies D. Systematic review: T-cell-based assays for the diagnosis of latent tuberculosis infection: an update. Ann Intern Med 149, 177–184 (2008). 18593687 10.7326/0003-4819-149-3-200808050-00241 PMC2951987

[b33] Bao Y. . Identification of IFN-gamma-producing innate B cells. Cell Res 24, 161–176 (2014). 24296781 10.1038/cr.2013.155 PMC3915900

[b34] Darwich L. . Secretion of interferon-gamma by human macrophages demonstrated at the single-cell level after costimulation with interleukin (IL)-12 plus IL-18. Immunology 126, 386–393 (2009). 18759749 10.1111/j.1365-2567.2008.02905.x PMC2669819

[b35] Wu U. I. & Holland S. M. Host susceptibility to non-tuberculous mycobacterial infections. Lancet Infect Dis 15, 968–980 (2015). 26049967 10.1016/S1473-3099(15)00089-4

[b36] Hassan S. S., Akram M., King E. C., Dockrell H. M. & Cliff J. M. PD-1, PD-L1 and PD-L2 Gene Expression on T-Cells and Natural Killer Cells Declines in Conjunction with a Reduction in PD-1 Protein during the Intensive Phase of Tuberculosis Treatment. PloS one 10, e0137646 (2015). 26359860 10.1371/journal.pone.0137646 PMC4567315

[b37] Griffith D. E. . Semiquantitative Culture Analysis during Therapy for Mycobacterium avium Complex Lung Disease. Am J Respir Crit Care Med 192, 754–760 (2015). 26068042 10.1164/rccm.201503-0444OC PMC4595680

[b38] Lam P. K. . Factors related to response to intermittent treatment of Mycobacterium avium complex lung disease. Am J Respir Crit Care Med 173, 1283–1289 (2006). 16514112 10.1164/rccm.200509-1531OC

[b39] Jeong B. H. . Intermittent antibiotic therapy for nodular bronchiectatic Mycobacterium avium complex lung disease. Am J Respir Crit Care Med 191, 96–103 (2015). 25393520 10.1164/rccm.201408-1545OC

[b40] Wallace R. J. Jr. . Macrolide/Azalide therapy for nodular/bronchiectatic mycobacterium avium complex lung disease. Chest 146, 276–282 (2014). 24457542 10.1378/chest.13-2538 PMC4694082

[b41] Tousif S. . T cells from Programmed Death-1 deficient mice respond poorly to Mycobacterium tuberculosis infection. PloS one 6, e19864 (2011). 21589883 10.1371/journal.pone.0019864 PMC3093409

[b42] Lazar-Molnar E. . Programmed death-1 (PD-1)-deficient mice are extraordinarily sensitive to tuberculosis. Proceedings of the National Academy of Sciences of the United States of America 107, 13402–13407 (2010). 20624978 10.1073/pnas.1007394107 PMC2922129

[b43] Butte M. J., Keir M. E., Phamduy T. B., Sharpe A. H. & Freeman G. J. Programmed death-1 ligand 1 interacts specifically with the B7-1 costimulatory molecule to inhibit T cell responses. Immunity 27, 111–122 (2007). 17629517 10.1016/j.immuni.2007.05.016 PMC2707944

[b44] Lee J. J., Chan A. & Tang T. Tuberculosis reactivation in a patient receiving anti-programmed death-1 (PD-1) inhibitor for relapsed Hodgkin’s lymphoma. Acta Oncol 55, 519–520 (2016). 26754959 10.3109/0284186X.2015.1125017

[b45] Sakai S. . CD4 T Cell-Derived IFN-gamma Plays a Minimal Role in Control of Pulmonary Mycobacterium tuberculosis Infection and Must Be Actively Repressed by PD-1 to Prevent Lethal Disease. PLoS Pathog 12, e1005667 (2016). 27244558 10.1371/journal.ppat.1005667 PMC4887085

[b46] Chang K. . Targeting the programmed cell death 1: programmed cell death ligand 1 pathway reverses T cell exhaustion in patients with sepsis. Crit Care 18, R3 (2014). 24387680 10.1186/cc13176 PMC4056005

[b47] Sampaio E. P. . Mycobacterium abscessus and M. avium trigger Toll-like receptor 2 and distinct cytokine response in human cells. Am J Respir Cell Mol Biol 39, 431–439 (2008). 18441280 10.1165/rcmb.2007-0413OC PMC2551704

[b48] Sweet L. & Schorey J. S. Glycopeptidolipids from Mycobacterium avium promote macrophage activation in a TLR2- and MyD88-dependent manner. Journal of leukocyte biology 80, 415–423 (2006). 16760377 10.1189/jlb.1205702

[b49] Wolfle S. J. . PD-L1 expression on tolerogenic APCs is controlled by STAT-3. Eur J Immunol 41, 413–424 (2011). 21268011 10.1002/eji.201040979

[b50] Loke P. & Allison J. P. PD-L1 and PD-L2 are differentially regulated by Th1 and Th2 cells. Proceedings of the National Academy of Sciences of the United States of America 100, 5336–5341 (2003). 12697896 10.1073/pnas.0931259100 PMC154346

[b51] Li M. . HBcAg induces PD-1 upregulation on CD4 + T cells through activation of JNK, ERK and PI3K/AKT pathways in chronic hepatitis-B-infected patients. Laboratory investigation; a journal of technical methods and pathology 92, 295–304 (2012). 22042085 10.1038/labinvest.2011.157

[b52] Griffith D. E. . Clinical and molecular analysis of macrolide resistance in Mycobacterium avium complex lung disease. Am J Respir Crit Care Med 174, 928–934 (2006). 16858014 10.1164/rccm.200603-450OC

[b53] Finnefrock A. C. . PD-1 blockade in rhesus macaques: impact on chronic infection and prophylactic vaccination. J Immunol 182, 980–987 (2009). 19124741 10.4049/jimmunol.182.2.980

[b54] Wang X. . ESAT-6 inhibits production of IFN-gamma by Mycobacterium tuberculosis-responsive human T cells. J Immunol 182, 3668–3677 (2009). 19265145 10.4049/jimmunol.0803579 PMC5488288

[b55] Snider G. L., Doctor L., Demas T. A. & Shaw A. R. Obstructive airway disease in patients with treated pulmonary tuberculosis. Am Rev Respir Dis 103, 625–640 (1971). 5579906 10.1164/arrd.1971.103.5.625

