# Supplementary material for: Attenuation of lymphocyte immune responses during Mycobacterium avium complex-induced lung disease due to increasing expression of programmed death-1 on lymphocytes
Source: Sci Rep. 2017 Feb 7;7:42004. doi: 10.1038/srep42004 (PMC5294633; doi:10.1038/srep42004)
Supplement: Supplementary Information [file srep42004-s1.docx]

**Attenuation of lymphocyte immune responses during *Mycobacterium avium* complex-induced lung disease due to increasing expression of programmed death-1 on lymphocytes**

Chin-Chung Shu, M.D.^1,2^; Jann-Yuan Wang, M.D., Ph.D.^3^; Ming-Fang Wu, Ph.D.^4,5^; Chen-Tu Wu M.D., Ph.D.^6^; Hsin-Chih Lai, Ph.D.^7^; Li-Na Lee, M.D., Ph.D.^3,8^; Bor-Luen Chiang, M.D., Ph.D.^9^; and Chong-Jen Yu, M.D., Ph.D.^3^;

**Correspondence to:**

Chong-Jen Yu, M.D., Ph.D.

Department of Internal Medicine

and

Bor-Luen Chiang, M.D., Ph.D.

Department of Medical Research

National Taiwan University Hospital

#7, Chung-Shan South Road, Taipei 100, Taiwan

E-mail: [jefferycjyu@ntu.edu.tw](mailto:jefferycjyu@ntu.edu.tw); gicmbor@ntu.edu.tw

Tel: 886-2-23562905; Fax: 886-2-23582867

**Running Title:** Attenuated Lymphocyte Immunity by increasing PD-1 in MAC-LD

**Conflict of Interest Disclosures from All Authors:** None

____________________________________

^1^Graduate Institute of Clinical Medicine and ^5^Toxicology, College of Medicine, National Taiwan University, Taipei, Taiwan

^2^Department of Traumatology, ^3^Department of Internal Medicine, ^6^Department of Pathology, ^8^Department of Laboratory Medicine, and ^9^Department of Pediatrics, National Taiwan University Hospital, Taipei, Taiwan

^4^Genomics Research Center, Academia Sinica, Taipei, Taiwan

^7^Department of Medical Biotechnology and Laboratory Science, Chang Gung University, Tao-Yuan, Taiwan

**Online Supplement File**

1. **The number of subject for the antigen stimulation assay of peripheral blood mononuclear cells.** The samples of all enrolled subjects underwent MAC MOI 100 and PHA stimulation, and the samples from 28/13 healthy controls, and 42/24 patients with MAC-lung disease (LD) underwent MAC MOI 20 and MAC sensitin stimulation assays, respectively.
2. **MAC bacilli stimulation induced the expression of the PD-1 ligand on macrophages**

The macrophages were derived from blood monocytes and stimulated by MAC bacilli for 2 days in order to measure the increase in PD-1 ligand expression. The PD-L1 and PD-L2 expressions were examined by flow cytometry (Fig. E5), and were significantly increased in the patients with MAC-LD after 2 days of MAC stimulation compared to mock stimulation.

1. **PD-1 and PD-L1 immuno-histochemical staining of MAC-infected lung tissue**

For immune-histochemical staining of PD-1 and PD-L1 expressions in the granulomatous lesions, 4 μm-thick sections from each formalin-fixed, paraffin-embedded tissue block were dewaxed with xylene and rehydrated through a graded series of ethanol. For PD-1 (Clone NAT105, Abcam Inc., Cambridge, MA, Cambridge, UK; dilution 1:50 for 30 min, antigen retrieval by a Leica Microsystem for 20 min at 100°C in Epitope Retrieval 2 solution, pH9.0) and PD-L1 (Proteintech Group Inc., Chicago, IL, USA; dilution 1:200 for 2 h, antigen retrieval by a Leica Microsystem for 40 min at 100°C in Epitope Retrieval 2 solution, pH9.0), a Leica Microsystem Bondmax autostainer (Leica Biosystems Newcastle Ltd., Newcastle upon Tyne, UK) was used according to the manufacturer’s instructions. The sections were then mounted, and PD-1 and PD-L1 were moderately stained in peri-granuloma lymphocytes and granuloma macrophages, respectively (Fig. E6).

**Table E1.** Cytokine production from peripheral blood mononuclear cells (PBMCs) stimulated by dead *Mycobacterium avium* bacilli for 48 hours by age group

|  |  | **Age <65 years**  **(n=58)** | |  | **Age ≥65 years**  **(n=22)** | |  |  |
| --- | --- | --- | --- | --- | --- | --- | --- | --- |
| **Cytokine** | Antigen | Mean | SD |  | Mean | SD | *p* value* |  |
| **TNF-α**  **(pg/ml)** | MAC MOI 20 | 858.8 | 1025.7 |  | 809.2 | 1316.7 | 0.978 | |
|  | MAC MOI 100 | 1772.2 | 2051.0 |  | 1910.1 | 3096.5 | 0.792 | |
|  | PHA | 2799.7 | 1662.5 |  | 3364.2 | 2066.2 | 0.232 | |
|  | LPS | 1086.0 | 803.2 |  | 1645.6 | 1503.4 | 0.141 | |
| **IFN-γ**  **(pg/ml)** | MAC MOI 20 | 49.7 | 70.6 |  | 35.9 | 50.4 | 0.507 | |
|  | MAC MOI 100 | 110.6 | 178.0 |  | 101.7 | 243.0 | 0.955 | |
|  | PHA | 5041.7 | 3981.4 |  | 6595.4 | 4515.2 | 0.142 | |

Abbreviations: IFN-γ, interferon-gamma; LPS, lipopolysaccharide; MAC, *Mycobacterium avium* complex; MOI, multiplicity of infection; PHA, phytohemaglutinin-L; TNF-α, tumor necrosis factor-alpha

**p* values by the Student’s *t* test.

**LEGEND**

**Figure E1.** In patients with *Mycobacterium avium* complex (MAC)-lung disease (LD) or controls (15 age- and sex-matched pairs), cytokine responses were measured by assaying peripheral blood mononuclear cells for 48 h with heat-killed MAC bacilli (multiplicity of infection [MOI]: 100) and phytohemaglutinin-L (PHA) (2.5 ng/ml). Values for tumor necrosis factor-alpha (TNF-α) and interferon-gamma (IFN-γ) are presented as dot plots with crossed line of mean values. Comparisons were preformed using the Mann Whitney *U* test. The TNF-α responses to MAC stimulation and PHA were 3873.3±34329.5 pg/ml (mean±standard deviation) and 4108.2±2858.9 pg/ml, respectively, in the healthy controls, and 952.1±1097.3 pg/ml and 2544.7±1155.9 pg/ml, respectively, in the patients with MAC-LD. The IFN-γ responses to MAC stimulation and PHA were 286.3±304.8 pg/ml and 6020.4±3898.1 pg/ml, respectively, in the healthy controls, and 96.3±138.1 pg/ml and 5186.6±4411.8 pg/ml, respectively, in the patients with MAC-LD.

**Figure E2**. Programmed cell death-1 (PD-1) expression in peripheral blood lymphocytes by flow cytometry in patients with *Mycobacterium avium* complex-lung disease (MAC-LD) and in age-matched healthy subjects (n=6 for CD3/CD8 and n=7 for CD4). Crossed lines denote mean values. Comparisons were performed using the Mann Whitney *U* test. *Represents 0.01≤p<0.05 and ** represents 0.001≤p<0.01 by the Mann Whitney *U* test. The PD-1 expression was higher in CD3 (mean±SD: 34.2±10.8% vs. 20.7±9.3%, p=0.023), CD4 (36.5±14.1% vs. 19.6±6.8%, p=0.004), CD8 (34.1±9.9% vs. 19.5±11.8%, p=0.043) in the patients with MAC-LD than in the age-matched healthy controls.

**Figure E3.** Cell subsets of peripheral blood leukocytes between the patients with *Mycobacterium avium* complex-lung disease (MAC-LD) and healthy subjects. **(A)** Dot plot and **(B)** bar chart (error bar: standard deviation) of the pooled results of flow cytometry (n=20). We discriminated lymphocytes and monocytes using forward scatter (FSC) and side scatter (SSC). We gated the lymphocyte markers CD3, CD19 and CD56 to identify lymphocytes (red circles, upper panel), and CD14 for monocytes (green circles, upper panel). CD4- and CD8-positive cells were gated in CD3-positive lymphocytes. Comparisons between healthy controls and patients with MAC-LD were performed using the Mann Whitney *U* test, and no significant inter-group differences were noted.

**Figure E4.** (A) Case demonstration of the expression of programmed cell death-1 (PD-1) on CD19, CD56 and CD4^+^CD25^+^ cells in peripheral blood lymphocytes. We focused on the lymphocyte population using forward scatter and side scatter. We gated CD3, CD4, CD19, and CD56 from the lymphocytes, and then gated CD25-positive cells from CD4 lymphocytes in nine healthy subjects and 12 patients with *Mycobacterium avium* complex-lung disease (MAC-LD). **(B)** Bar chart (error bar: standard deviation), and **(C)** histogram by flow cytometry of the PD-1 expression on CD4^+^CD25^+^ cells. Comparisons between healthy controls and patients with MAC-LD were performed using the Mann Whitney *U* test. *0.01≤*p*<0.05; MFI, mean fluorescence intensity.

**Figure E5.** The percentage of programmed death-ligand 1 (PD-L1) and PD-L2 in blood monocyte-derived macrophages after MAC bacilli (MOI 100) or mock stimulation for 48 h in patients with *Mycobacterium avium* complex-lung disease (MAC-LD). Crossed lines denote mean values. Comparisons of mock and MAC stimulation in the same subject were performed using the Wilcoxon test for pairs of comparisons. *0.01<p<0.05. The expressions of PD-L1 (4.8±3.7% vs. 1.3±1.2%, p=0.016) and PD-L2 (2.0±3.0% vs. 0.6±0.6%, p=0.031) were higher in with MAC stimulation than with mock stimulation.

**Figure E6.** High power field photomicrographs showing **(A)** PD-1 reactivity in lymphocytes around granulomas, and **(B)** PD-L1 reactivity in the aggregated macrophages within granulomas of paraformaldehyde-fixed lung tissue in a patient with *Mycobacterium avium* complex-lung disease (MAC-LD).

**Figure E7**. Programmed cell death ligand-1 (PD-L1) expressions in peripheral blood lymphocytes and monocytes by flow cytometry. Case demonstration is shown. We identified lymphocytes (red circles) and monocytes (green circles) using forward scatter (FSC) and side scatter (SSC) (upper panel). We gated the monocytes using the marker CD14, and measured the expression of PD-L1. We also gated the lymphocytes using FSC/SSC, and measured the expression of PD-L1. MAC, *Mycobacterium avium* complex; LD, lung disease.

**Figure E8** The expressions of programmed cell death-1 (PD-1) on CD3, CD4 and CD8 T lymphocytes were measured (A) before and (B) after 2 months of treatment for *Mycobacterium avium* complex lung disease (MAC-LD) as demonstrated by dot plot in one case. We discriminated lymphocytes using forward scatter (FSC) and side scatter (SSC). We gated CD3 in the lymphocytes, and then gated CD4 and CD8 in CD3-positive lymphocytes and further measured PD-1 in each lymphocyte subgroup.

**Figure E9**. Cytokine production was measured in a PBMC stimulation assay using *Mycobacterium avium* complex (MAC [MOI:100]) with blocking antibodies for programmed cell death-1 (PD-1) and programmed death ligand-1&2 (PD-L1&2). The post-blocking cytokine levels were compared between the controls and patients with MAC*-*lung disease (LD). Crossed line represent mean values. The expressions of tumor necrosis factor-alpha (TNF-α) were 2964.1±2627.2 pg/ml (mean±standard deviation) vs. 2463.2±1412.6 pg/ml (p=1.000, Mann-Whitney U test), and for IFN-γ 582.1±595.8 vs. 39.0±19.1 pg/ml (p=0.109, Mann-Whitney U test). The degree of increase after the blocking assay for TNF-α was 1.4±0.5 times in the controls and 1.5±0.8 times in the MAC-LD group (p=0.661, Mann-Whitney U test), and for IFN-γ 1.4±0.4 (controls) vs. 1.7±0.9 times (patients) (p=0.475, Mann-Whitney U test).

**Figure E10**. After assaying human monocyte-derived macrophages with heat-killed *Mycobacterium avium* bacilli (MAC [MOI 100]) for 24 hours, we co-cultured the macrophages with autologous CD14-negative cells for 5 days. Interferon-gamma (IFN-γ) was measured using antagonizing antibodies for programmed cell death 1(PD-1), and PD ligand-1 (PD-L1) during coculture. Post-blocking IFN-γ levels were compared between the healthy controls (HC) and patients with MAC-lung disease (LD). Crossed lines represent mean values. The Mann-Whitney *U* test was used to compare the controls and patients. The expression of IFN-γ after blocking PD-1/PD-L1 in the control group (828.2±758.7 pg/ml [mean±standard deviation]) was not significantly different from that in the patients with MAC-LD (284.1±115.5 pg/ml) (p=0.142, Mann Whitney U test). The degree of increase in IFN-γ after blocking PD-1/PD-L1 was similar between the controls and patients (2.8±1.9 vs. 3.0±1.2, p=0.852, Mann Whitney U test).

**Figure E11.** Human blood monocyte derived macrophages were stimulated with *Mycobacterium avium* complex (MAC) (MOI 100) and then co-cultured with autologous lymphocytes for 5 days. We re-stimulated the cells with anti-CD3 and anti-CD28 antibodies for 1 day and measured (A) the percentage of interferon-gamma (IFN-γ) expressing CD4 lymphocytes, and (B) the percentage of programmed death-1 (PD-1) expressed on CD4+IFN-γ+ cells by flow cytometry in six controls and eight patients with MAC-lung disease (LD). The values and error bars show means and standard deviations, respectively. The Mann-Whitney U test was used for inter-group comparisons. The PD-1 expression was higher on CD4^+^IFN-γ^+^ lymphocytes in the patients with MAC-LD than in the controls (65.7±17.2% vs. 44.4±19.4%, p=0.044). (C) The percentages of PD-1 and PD-1 ligand 1 (PD-L1) on CD4 lymphocytes were compared between those with or without treatment of the antagonizing PD-1/PD-L1 antibodies before the co-culture assay using the Wilcoxon test (n=11). The expression of PD-1 decreased from 35.5±24.6% (mean ± standard deviation) to 33.0±24.9% (p=0.041, Wilcoxon test), and PD-L1 decreased from 22.0±21.9% to 7.8±7.7% (p=0.021, Wilcoxon test). The error bars indicate the standard deviation. The changes in percentage were 7.04% and 64.54% for PD-1 and PD-L1, respectively.

**Figure E12**. We examined the cytokine responses with different multiplicities of infection (MOI) in the healthy controls (n=11) by assaying peripheral blood mononuclear cells with heat-killed bacilli of *Mycobacterium avium* subspecies. (A) Tumor necrosis factor-alpha (TNF-α); and (B) interferon-gamma, (IFN-γ). The values and error bars show means and standard deviations, respectively. Comparisons were performed using the Wilcoxon test. NC, negative control; PHA, phytohemaglutinin-L.

**Figure E1.** In patients with *Mycobacterium avium* complex (MAC)-lung disease (LD) or controls (15 age- and sex-matched pairs), cytokine responses were measured by assaying peripheral blood mononuclear cells for 48 h with heat-killed MAC bacilli (multiplicity of infection [MOI]: 100) and phytohemaglutinin-L (PHA) (2.5 ng/ml). Values for tumor necrosis factor-alpha (TNF-α) and interferon-gamma (IFN-γ) are presented as dot plots with crossed line of mean values. Comparisons were preformed using the Mann Whitney *U* test. The TNF-α responses to MAC stimulation and PHA were 3873.3±34329.5 pg/ml (mean±standard deviation) and 4108.2±2858.9 pg/ml, respectively, in the healthy controls, and 952.1±1097.3 pg/ml and 2544.7±1155.9 pg/ml, respectively, in the patients with MAC-LD. The IFN-γ responses to MAC stimulation and PHA were 286.3±304.8 pg/ml and 6020.4±3898.1 pg/ml, respectively, in the healthy controls, and 96.3±138.1 pg/ml and 5186.6±4411.8 pg/ml, respectively, in the patients with MAC-LD.


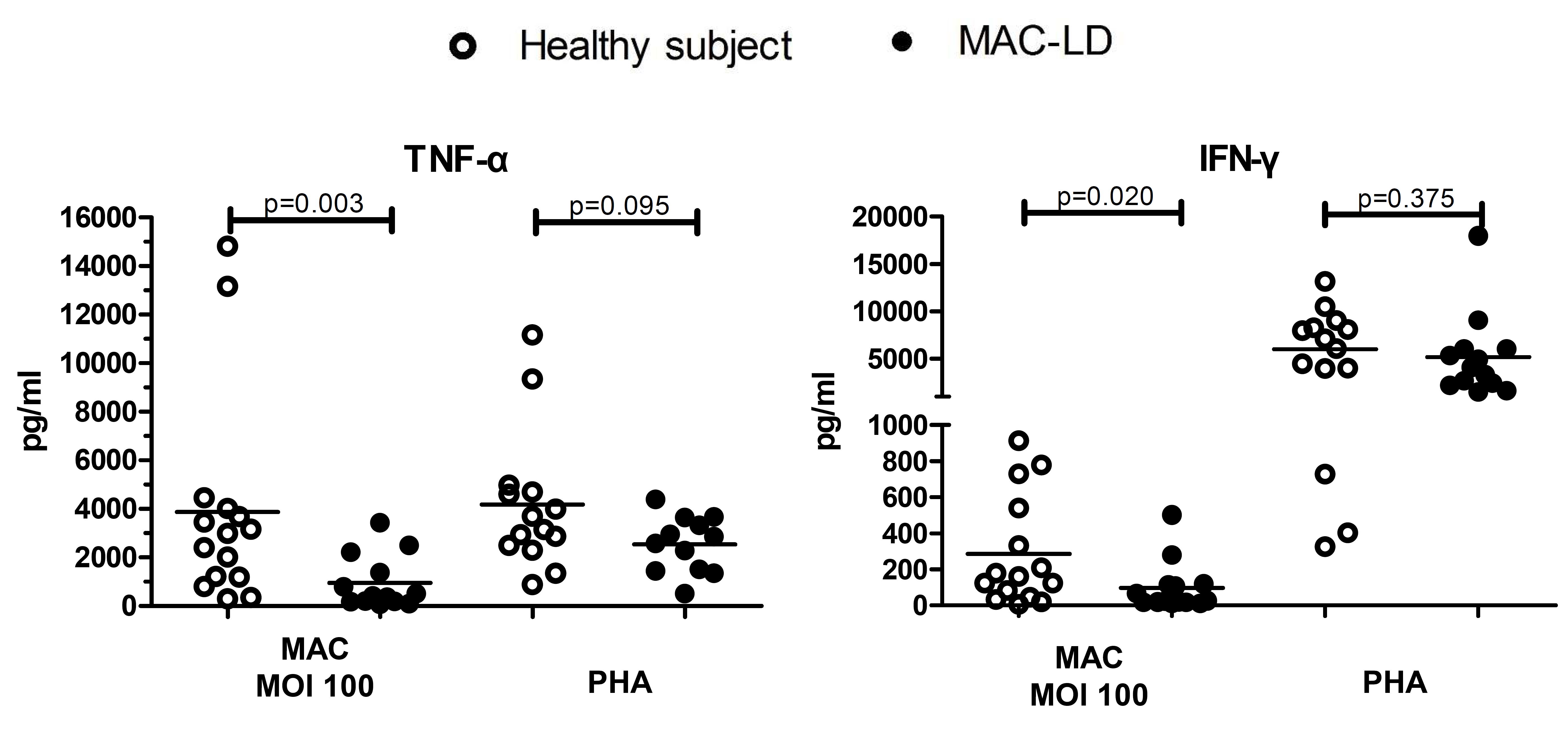


**Figure E2**. Programmed cell death-1 (PD-1) expression in peripheral blood lymphocytes by flow cytometry in patients with *Mycobacterium avium* complex-lung disease (MAC-LD) and in age-matched healthy subjects (n=6 for CD3/CD8 and n=7 for CD4). Crossed lines denote mean values. Comparisons were performed using the Mann Whitney *U* test. *Represents 0.01≤p<0.05 and ** represents 0.001≤p<0.01 by the Mann Whitney *U* test. The PD-1 expression was higher in CD3 (mean±SD: 34.2±10.8% vs. 20.7±9.3%, p=0.023), CD4 (36.5±14.1% vs. 19.6±6.8%, p=0.004), CD8 (34.1±9.9% vs. 19.5±11.8%, p=0.043) in the patients with MAC-LD than in the age-matched healthy controls.


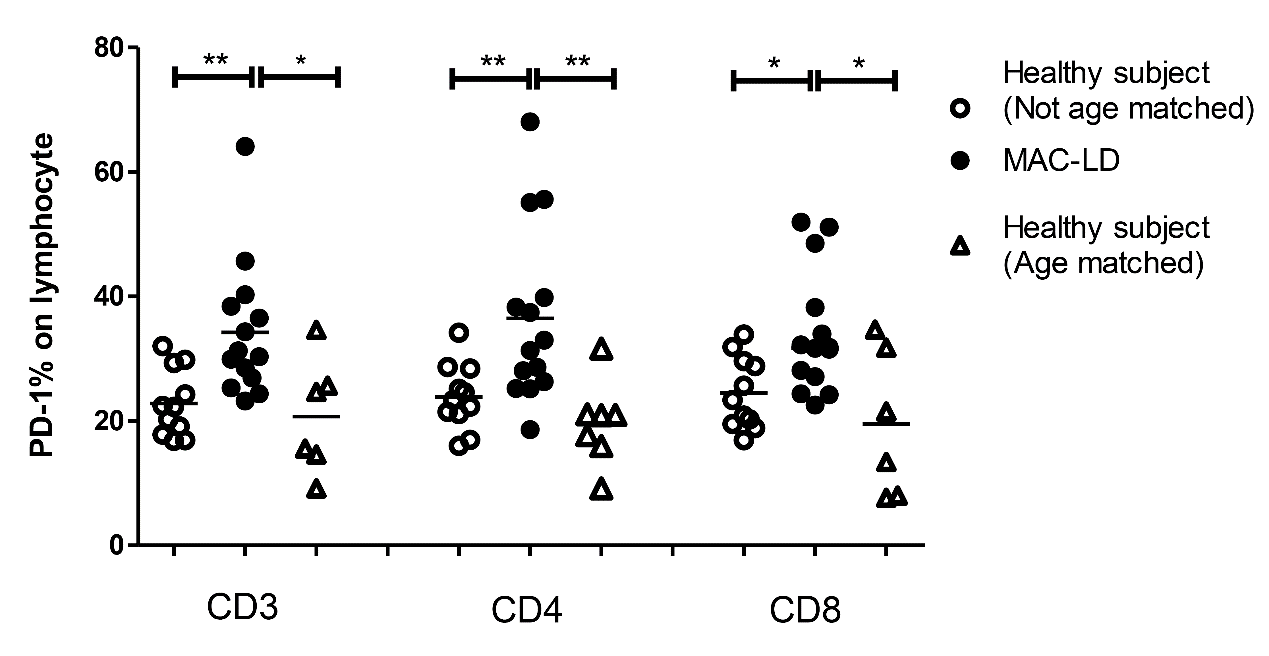


**Figure E3.** Cell subsets of peripheral blood leukocytes between the patients with *Mycobacterium avium* complex-lung disease (MAC-LD) and healthy subjects. **(A)** Dot plot and **(B)** bar chart (error bar: standard deviation) of the pooled results of flow cytometry (n=20). We discriminated lymphocytes and monocytes using forward scatter (FSC) and side scatter (SSC). We gated the lymphocyte markers CD3, CD19 and CD56 to identify lymphocytes (red circles, upper panel), and CD14 for monocytes (green circles, upper panel). CD4- and CD8-positive cells were gated in CD3-positive lymphocytes. Comparisons between healthy controls and patients with MAC-LD were performed using the Mann Whitney *U* test, and no significant inter-group differences were noted.(A)

Healthy subjects MAC-LD patients


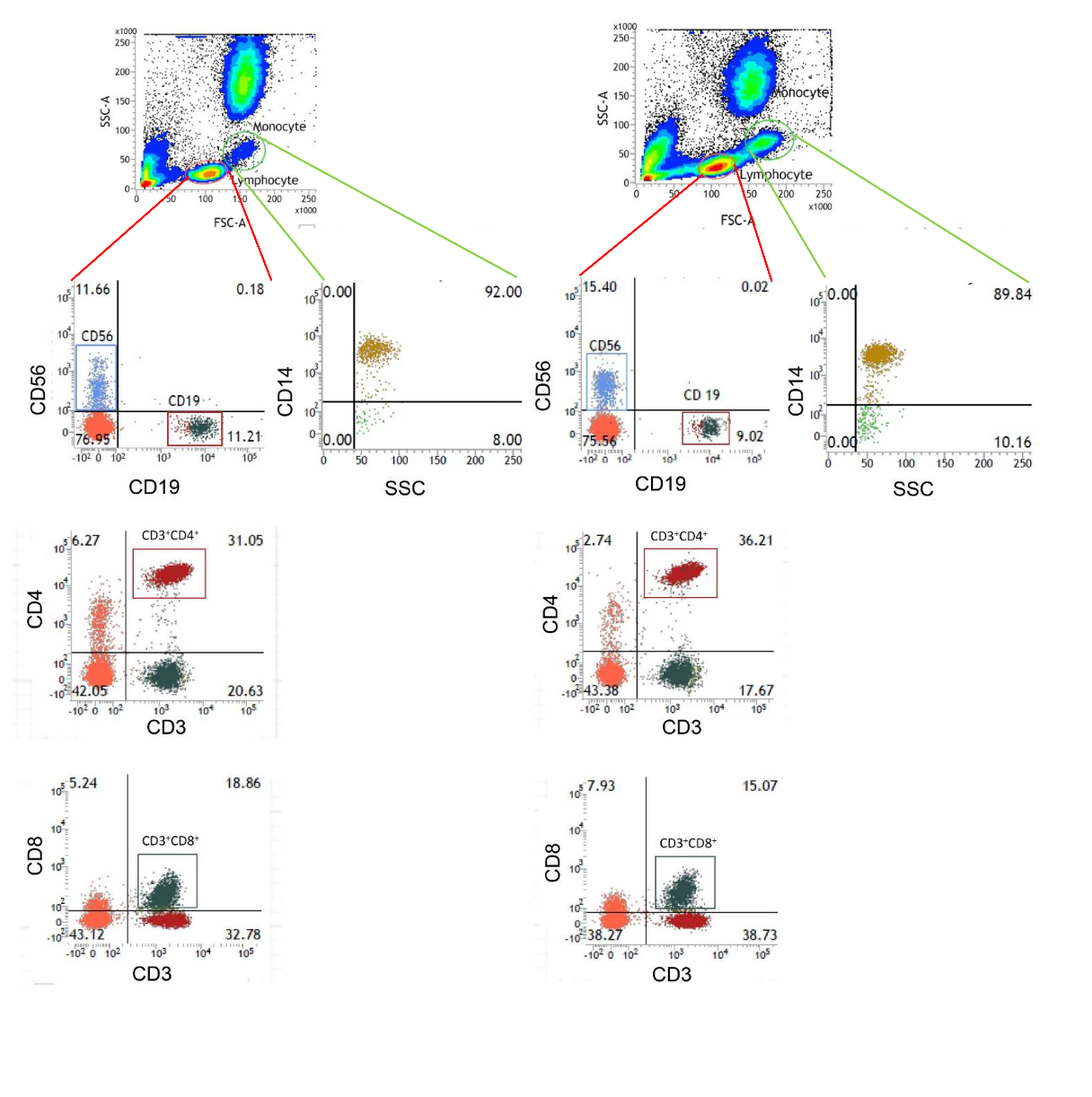
(B)

**Figure E4.** (A) Case demonstration of the expression of programmed cell death-1 (PD-1) on CD19, CD56 and CD4^+^CD25^+^ cells in peripheral blood lymphocytes. We focused on the lymphocyte population using forward scatter and side scatter. We gated CD3, CD4, CD19, and CD56 from the lymphocytes, and then gated CD25-positive cells from CD4 lymphocytes in nine healthy subjects and 12 patients with *Mycobacterium avium* complex-lung disease (MAC-LD). **(B)** Bar chart (error bar: standard deviation), and **(C)** histogram by flow cytometry of the PD-1 expression on CD4^+^CD25^+^ cells. Comparisons between healthy controls and patients with MAC-LD were performed using the Mann Whitney *U* test. *0.01≤*p*<0.05; MFI, mean fluorescence intensity.

(A)

(B)

(C)

Healthy subjects MAC-LD patients


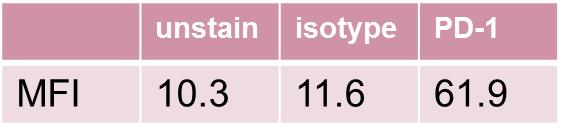

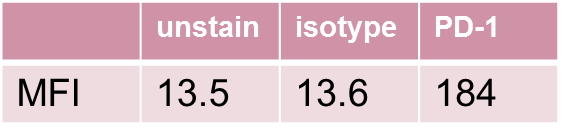

**Figure E5.** The percentage of programmed death-ligand 1 (PD-L1) and PD-L2 in blood monocyte-derived macrophages after MAC bacilli (MOI 100) or mock stimulation for 48 h in patients with *Mycobacterium avium* complex-lung disease (MAC-LD). Crossed lines denote mean values. Comparisons of mock and MAC stimulation in the same subject were performed using the Wilcoxon test for pairs of comparisons. *0.01<p<0.05. The expressions of PD-L1 (4.8±3.7% vs. 1.3±1.2%, p=0.016) and PD-L2 (2.0±3.0% vs. 0.6±0.6%, p=0.031) were higher in with MAC stimulation than with mock stimulation.

**Figure E6.** High power field photomicrographs showing **(A)** PD-1 reactivity in lymphocytes around granulomas, and **(B)** PD-L1 reactivity in the aggregated macrophages within granulomas of paraformaldehyde-fixed lung tissue in a patient with *Mycobacterium avium* complex-lung disease (MAC-LD).


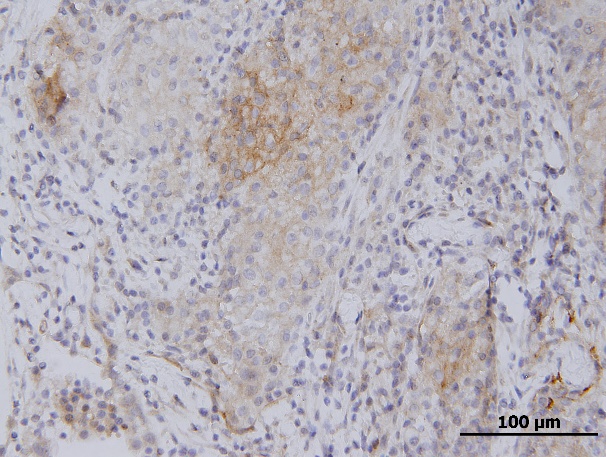

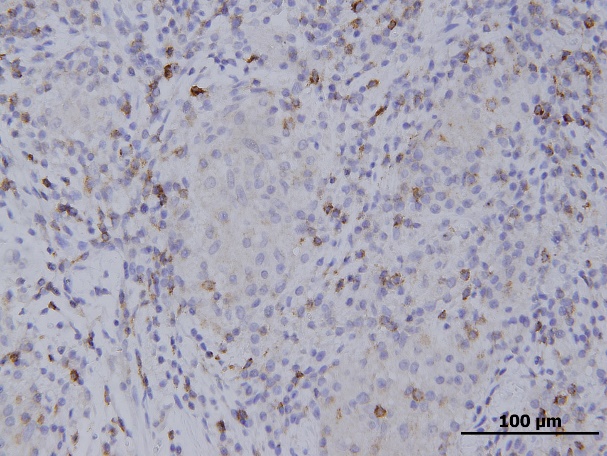
(A) (B)

**Figure E7**. Programmed cell death ligand-1 (PD-L1) expressions in peripheral blood lymphocytes and monocytes by flow cytometry. Case demonstration is shown. We identified lymphocytes (red circles) and monocytes (green circles) using forward scatter (FSC) and side scatter (SSC) (upper panel). We gated the monocytes using the marker CD14, and measured the expression of PD-L1. We also gated the lymphocytes using FSC/SSC, and measured the expression of PD-L1. MAC, *Mycobacterium avium* complex; LD, lung disease.


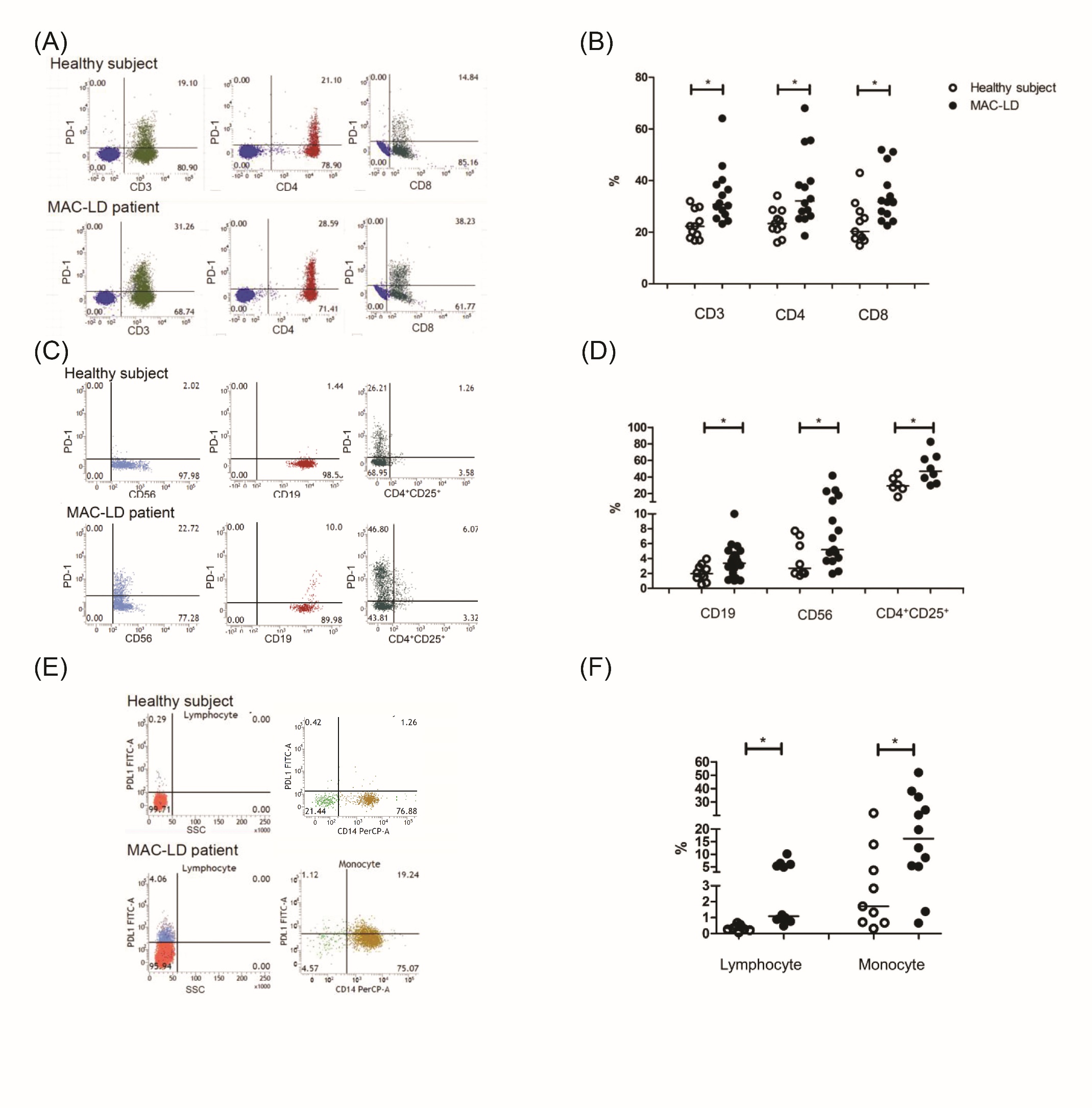

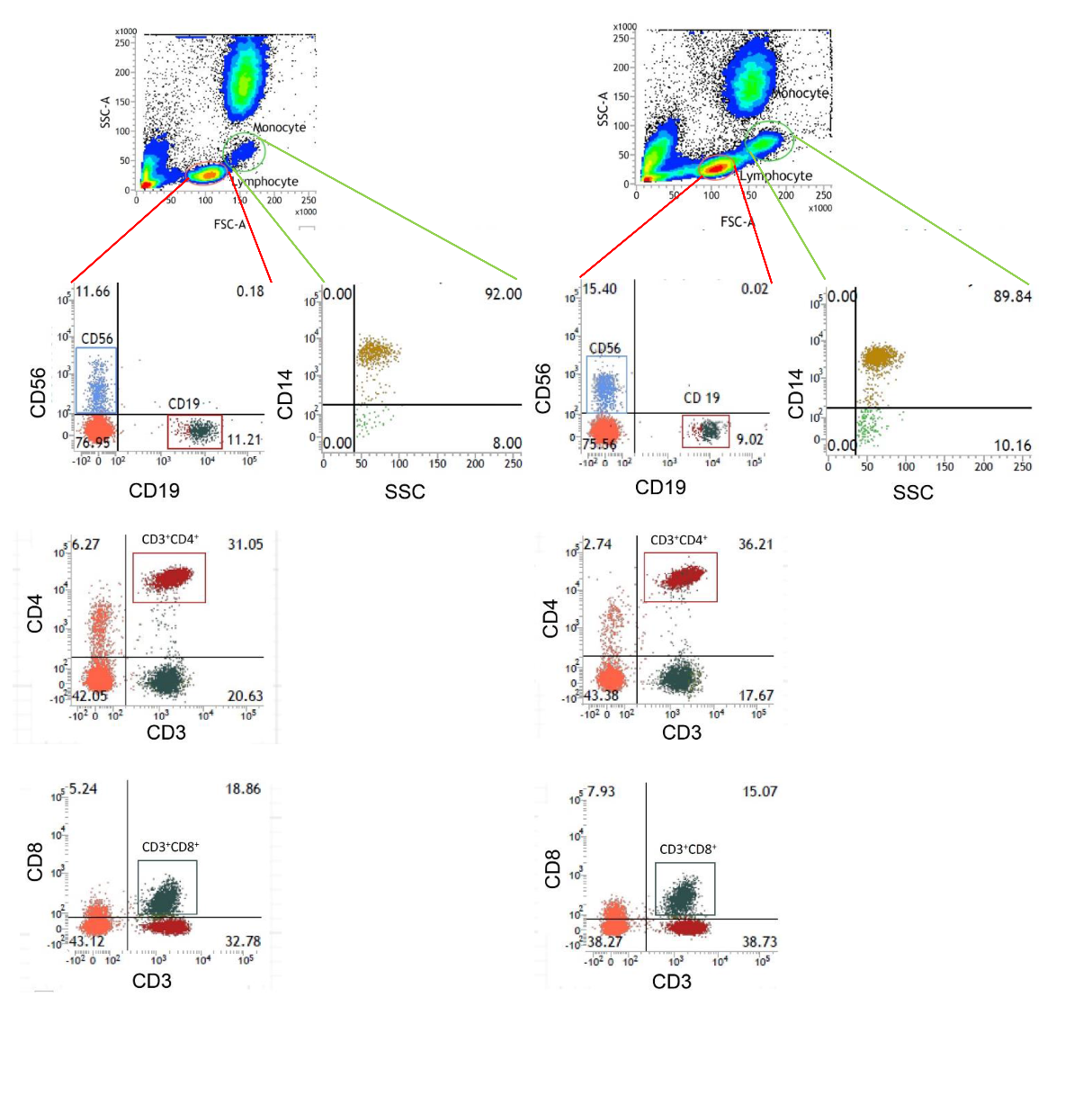


**Figure E8**. The expressions of programmed cell death-1 (PD-1) on CD3, CD4 and CD8 T lymphocytes were measured (A) before and (B) after 2 months of treatment for *Mycobacterium avium* complex lung disease (MAC-LD) as demonstrated by dot plot in one case. We discriminated lymphocytes using forward scatter (FSC) and side scatter (SSC). We gated CD3 in the lymphocytes, and then gated CD4 and CD8 in CD3-positive lymphocytes and further measured PD-1 in each lymphocyte subgroup.


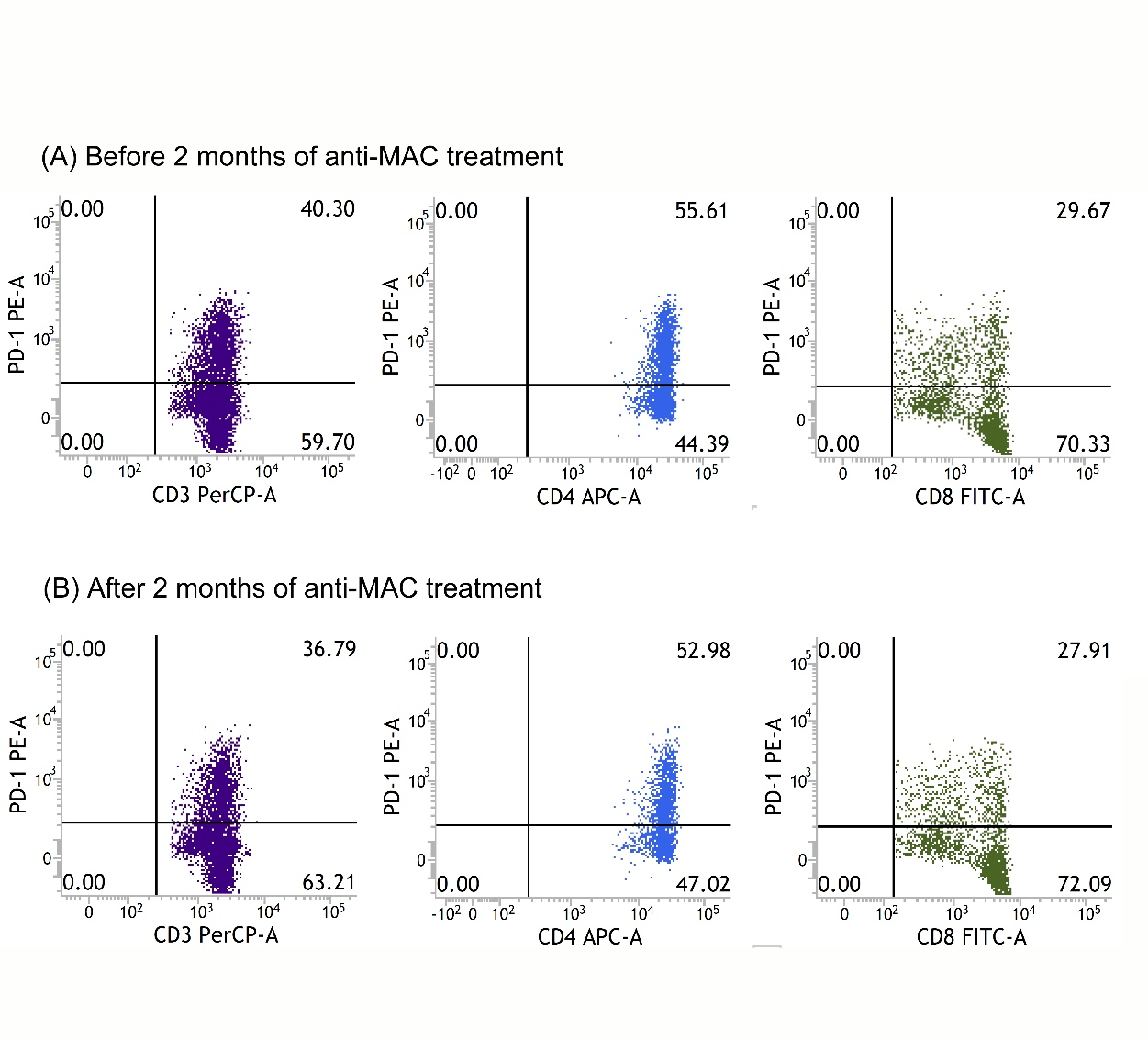


**Figure E9**. Cytokine production was measured in a PBMC stimulation assay using *Mycobacterium avium* complex (MAC [MOI:100]) with blocking antibodies for programmed cell death-1 (PD-1) and programmed death ligand-1&2 (PD-L1&2). The post-blocking cytokine levels were compared between the controls and patients with MAC*-*lung disease (LD). Crossed lines represent mean values. The expressions of tumor necrosis factor-alpha (TNF-α) were 2964.1±2627.2 pg/ml (mean±standard deviation) vs. 2463.2±1412.6 pg/ml (p=1.000, Mann-Whitney U test), and for IFN-γ 582.1±595.8 vs. 39.0±19.1 pg/ml (p=0.109, Mann-Whitney U test). The degree of increase after the blocking assay for TNF-α was 1.4±0.5 times in the controls and 1.5±0.8 times in the MAC-LD group (p=0.661, Mann-Whitney U test), and for IFN-γ 1.4±0.4 (controls) vs. 1.7±0.9 times (patients) (p=0.475, Mann-Whitney U test).

**Figure E10.** After assaying human monocyte-derived macrophages with heat-killed *Mycobacterium avium* bacilli (MAC [MOI 100]) for 24 hours, we co-cultured the macrophages with autologous CD14-negative cells for 5 days. Interferon-gamma (IFN-γ) was measured using antagonizing antibodies for programmed cell death 1(PD-1), and PD ligand-1 (PD-L1) during coculture. Post-blocking IFN-γ levels were compared between the healthy controls (HC) and patients with MAC-lung disease (LD). Crossed lines represent mean values. The Mann-Whitney *U* test was used to compare the controls and patients. The expression of IFN-γ after blocking PD-1/PD-L1 in the control group (828.2±758.7 pg/ml [mean±standard deviation]) was not significantly different from that in the patients with MAC-LD (284.1±115.5 pg/ml) (p=0.142, Mann Whitney U test). The degree of increase in IFN-γ after blocking PD-1/PD-L1 was similar between the controls and patients (2.8±1.9 vs. 3.0±1.2, p=0.852, Mann Whitney U test).

**Figure E11.** Human blood monocyte derived macrophages were stimulated with *Mycobacterium avium* complex (MAC) (MOI 100) and then co-cultured with autologous lymphocytes for 5 days. We re-stimulated the cells with anti-CD3 and anti-CD28 antibodies for 1 day and measured (A) the percentage of interferon-gamma (IFN-γ) expressing CD4 lymphocytes, and (B) the percentage of programmed death-1 (PD-1) expressed on CD4+IFN-γ+ cells by flow cytometry in six controls and eight patients with MAC-lung disease (LD). The values and error bars show means and standard deviations, respectively. The Mann-Whitney U test was used for inter-group comparisons. The PD-1 expression was higher on CD4^+^IFN-γ^+^ lymphocytes in the patients with MAC-LD than in the controls (65.7±17.2% vs. 44.4±19.4%, p=0.044). (C) The percentages of PD-1 and PD-1 ligand 1 (PD-L1) on CD4 lymphocytes were compared between those with or without treatment of the antagonizing PD-1/PD-L1 antibodies before the co-culture assay using the Wilcoxon test (n=11). The expression of PD-1 decreased from 35.5±24.6% (mean ± standard deviation) to 33.0±24.9% (p=0.041, Wilcoxon test), and PD-L1 decreased from 22.0±21.9% to 7.8±7.7% (p=0.021, Wilcoxon test). The error bars indicate the standard deviation. The changes in percentage were 7.04% and 64.54% for PD-1 and PD-L1, respectively

(A) (B)

(C)

**Figure E12**. We examined the cytokine responses with different multiplicities of infection (MOI) in the healthy controls (n=11) by assaying peripheral blood mononuclear cells with heat-killed bacilli of *Mycobacterium avium* subspecies. (A) Tumor necrosis factor-alpha (TNF-α); and (B) interferon-gamma, (IFN-γ). The values and error bars show means and standard deviations, respectively. Comparisons were performed using the Wilcoxon test. NC, negative control; PHA, phytohemaglutinin-L.

1. (B)
